# Application of t-intuitionistic fuzzy subgroup to Sylow theory

**DOI:** 10.1016/j.heliyon.2023.e19822

**Published:** 2023-09-09

**Authors:** Laila Latif, Umer Shuaib

**Affiliations:** a Department of Mathematics, Government College University Faisalabad, 38000, Pakistan

**Keywords:** t-Intuitionistic fuzzy subgroup (t-IFSG), t-Intuitionistic fuzzy conjugate element, t-Intuitionistic fuzzyp− Subgroup, t-Intuitionistic fuzzy conjugate subgroup (t-IFCSG), t-Intuitionistic fuzzy Sylowp−, Subgroup ($t-IF{Syl}_{p}$)

## Abstract

In this paper, we define the notion of a t-intuitionistic fuzzy conjugate element and determine the t-intuitionistic fuzzy conjugacy classes of a t-intuitionistic fuzzy subgroup. We propose the idea of a t-intuitionistic fuzzy p− subgroup and prove the t-intuitionistic fuzzy version of the Cauchy theorem. In addition, we present the idea of a t-intuitionistic fuzzy conjugate subgroup and investigate various fundamental algebraic characteristics of this notion. Furthermore, we provide the idea of the t-intuitionistic fuzzy Sylow p− subgroup and prove the t-intuitionistic fuzzification of Sylow's theorems.

Mathematics Subject Classification: 08A72, 20N25, 03E72.

## 1 Introduction

The Sylow theorems are an important part of finite group theory, and they have many important uses in classical group theory, especially when it comes to classifying the finite simple group. Sylow applied this theory in the framework of solving an algebraic equation and associated the roots of this equation to the solvability of its Galois group by radicals. One of the main advantages of these theorems is that they establish a partial converse of Lagrange's Theorem in the literature. Moreover, one can easily investigate the existence of a subgroup of certain orders utilizing this technique.

Fuzzy approaches have several advantages over crisp ones: the most important being that they have more flexible decision boundaries and are thus characterized by their higher ability to adjust to a specific domain of application and more accurately reflect its particularities. The importance of fuzzy logic derives from the fact that most modes of human reasoning, especially commonsense reasoning, are approximate in nature. The concept of an IFS is characterized by membership and non-membership functions as much efficient way to cope with uncertainty. The concept of IFS is very useful in providing a flexible model to elaborate on the uncertainty and vagueness involved in decision-making. It is a very useful tool for human consistency in reasoning under imperfectly defined facts and imprecise knowledge.

The concept of fuzzy set (FS) theory was introduced by Zadeh [1]. Rosenfeld [2] initiated the idea of a fuzzy subgroup(FSG) and established numerous essential characteristics of this phenomenon. In Ref. [3], Sunderrajan initiated the study of homomorphism and anti-homomorphism of L-fuzzy quotient ℓ-groups. Atanassov [4,5] proposed the study of IFS in 1986. In Ref. [6], Ejegwa et al. presented a concise overview of IFS. This theory has received a great deal of attention from researchers in the last two decades as it has been effectively applied in career determination [7,8], pattern recognition [9,10], medical diagnosis [11,12], expert systems [13,14], neural networks [15,16], decision making [17,18], machine learning [19,20], and semantic representations [21,22]. A useful approach was developed to study intuitionistic fuzzy soft rough sets based on decision-making in Refs. [23,24]. Biswas [25] proposed the idea of intuitionistic fuzzy subgroup (IFSG). In Ref. [26], Ahn et al. discussed various types of sublattices of the lattice of intuitionistic fuzzy subgroups. Sharma [27] proved many properties of IFSG. For more development on IFSG, we refer to Refs. [[28], [29], [30], [31], [32]]. In addition, the ideas of an intuitionistic fuzzy ring [33], an intuitionistic fuzzy ideal [34], an intuitionistic fuzzy normed ring [35], an intuitionistic fuzzy soft ring [36], and an intuitionistic fuzzy normal subring [37] were proposed. The theory of complex IFSG was proposed and developed by Husban et al. [38,39]. The theory of t-intuitionistic fuzzy subgroup(t-IFSG) was presented by Sharma in Ref. [40]. Moreover, a comprehensive development of the theory of t-IFSG can be viewed in Refs. [41,42]. In Ref. [43], Kattan et al. proved the interval-valued intuitionistic fuzzy version of Lagrange's theorem.

Real-world data can be challenging due to its imprecision, resulting from uncertainties, vagueness, or conflicting information. Although traditional approaches like as FS and IFS are often used in the management of imprecision. The t-IFS theory provides an additional parameterizing component, which makes it possible to express uncertain data in a more comprehensive manner. This parameter, which is denoted by the letter "t" is essential in defining the degree of imprecision that is taken into account during modeling. Analysts can more precisely depict imprecision by modulating the impact of inconsistent or vague information via "t" resulting in more reliable outcomes and decision-making. This adaptability is essential when dealing with varying degrees of imprecision. Consequently, t-IFS enhances the precision of managing imprecise data. In contrast to the theories of classical FS and IFS, it is an essential tool for addressing data imprecision by introducing a parameterizing factor during the process. It is a very useful technique as it provides a flexible model to counter the uncertainty and vagueness involved in making decisions.

The following discussion describes the motivations of this present study.

• Introduce t-intuitionistic fuzzy conjugate elements and conjugacy classes (in Section 3). These phenomena help study t-IFSG structure and organization. Analyzing conjugate elements' patterns, connections, and symmetries may reveal a subgroup's structure. Classifying and characterizing t-IFSG is made easier using conjugate elements and their conjugacy classes. These tools help organize elements into subgroups based on their conjugacy, which can provide insight into common properties, patterns, and behaviors.
• Introduce the notion of the t-intuitionistic fuzzy p-subgroup and prove the t-intuitionistic fuzzy version of the Cauchy theorem (in Section 4). They are critical for classifying finite groups. It assists researchers in determining the likely order of components inside a group, which may subsequently be used to organize the groupings based on their attributes. This categorization aids in the study of qualities by allowing for a better understanding of the similarities and differences between distinct groups.
• Initiate the ideas of t-intuitionistic fuzzy conjugate subgroups and their classes (in Section 5). These concepts help classify and organize subgroups within a larger group. The study of groups is simplified when subgroups are divided into discrete classes based on shared qualities or characteristics. This categorization is used to establish significant theorems, such as Sylow's theorems, which reveal information about the structure of finite groups. These ideas are strongly related to normal subgroups.
• Explore the idea of a t-intuitionistic fuzzy Sylow p− subgroup and prove a t-intuitionistic fuzzy version of Sylow's theorems (in Section 6). To better understand how subgroups work within finite groups, we can use t-intuitionistic logic to fuzzify Sylow's theorem. These are helpful even when dealing with uncertainties and imprecisions in real-life situations. The t-intuitionistic fuzzy version of Sylow's theorem can be applied to practical problems involving uncertainty, vagueness, and imprecision in subgroup analysis. This framework can improve decision-making, data analysis, optimization, and social network analysis. It helps to understand better real-life scenarios that have uncertain group structures.

The remaining sections of the article are arranged in the following order: Section 1 presents the introduction and motivation; Section 2 contains some elementary definitions and results; and conclusion and future scopes are presented in Section 7.

## 2 Preliminaries

This section contains some basic definitions and results related to them, which are very important to recognize the modernity of this article.

Definition 1 [40] A t-intuitionistic fuzzy set (t-IFS) $A_{t}$ of a universal set U is defined as:

$$A_{t}=\left\{\left(a_{1},\mu_{A_{t}}\left(a_{1}\right),\nu_{A_{t}}\left(a_{1}\right)\right):a_{1}\in U\right\},t\in \left[0,1\right]$$

where $\mu_{A_{t}}\left(a_{1}\right)$ and $\nu_{A_{t}}\left(a_{1}\right)$ are functions that assign degrees of membership and non-membership, respectively, to the element $a_{1}$ in U. The functions $\mu_{A_{t}}\left(a_{1}\right)$ and $\nu_{A_{t}}\left(a_{1}\right)$ are defined based on an existing IFS $A=\left(\mu_{A}\left(a_{1}\right),\nu_{A}\left(a_{1}\right)\right)$ on U. Specifically,

$$\mu_{A_{t}}\left(a_{1}\right)=min\;{\{\mu }_{A}\left(a_{1}\right),t\}\;\text{and }\;\nu_{A_{t}}\left(a_{1}\right)=max\left\{\nu_{A}\left(a_{1}\right),1-t\right\}\text{.}$$

The condition is true for each $a_{1}$ in U, $0\leq \mu_{A_{t}}\left(a_{1}\right)+\nu_{A_{t}}\left(a_{1}\right)\leq 1$.

Definition 2 [40] A $t-IFS\;A_{t}$ is called t-intuitionistic fuzzy subgroup$\left(t-IFSG\right)\;A_{t}$ of group G if it admits the following conditions:

1) $\mu_{A_{t}}\left(a_{1}b_{1}\right)\geq min\left\{\mu_{A_{t}}\left(a_{1}\right),\mu_{A_{t}}\left(b_{1}\right)\right\}$.
2) $\nu_{A_{t}}\left(a_{1}b_{1}\right)\leq max\left\{\nu_{A_{t}}\left(a_{1}\right),\nu_{A_{t}}\left(b_{1}\right)\right\}$.
3) $\mu_{A_{t}}\left(a_{1}^{-1}\right)=\mu_{A_{t}}\left(a_{1}\right)$.
4) $\nu_{A_{t}}\left(a_{1}^{-1}\right)=\nu_{A_{t}}\left(a_{1}\right)$, $\forall a_{1},b_{1}\in G$.

In $A_{t}\text{,}$ the terms $\mu_{A_{t}}\left(a_{1}b_{1}\right),\mu_{A_{t}}\left(a_{1}\right)$ and $\mu_{A_{t}}\left(b_{1}\right)$ represent the degree of membership of the elements $a_{1}b_{1},a_{1},b_{1}\text{.}$ On the other hand, the expressions $\nu_{A_{t}}\left(a_{1}b_{1}\right),\nu_{A_{t}}\left(a_{1}\right)$ and $\nu_{A_{t}}\left(b_{1}\right)$ indicate the degree of non-membership degrees to the same elements in $A_{t}$.

Definition 3 [41] Let $A_{t}$ be a t−IFS of a universe U. The (ρ,η)− cut set of t−IFS is a subset of the universe U, which may be defined as:

$${ℭ}_{\left(\rho ,\eta \right)}\left(A_{t}\right)=\left\{a_{1}\in U:\mu_{A_{t}}\left(a_{1}\right)\geq \rho \;\text{and}\;\nu_{A_{t}}\left(a_{1}\right)\leq \eta \right\}$$

where 0≤ρ≤1 and 0≤η≤1 such that 0≤ρ+η≤1.

Where $\mu_{A_{t}}\left(a_{1}\right)$ and $\nu_{A_{t}}\left(a_{1}\right)$ indicate the membership and non-membership degrees to the element $a_{1}$ in $A_{t}$.

Definition 4 [41] The support set $A_{t}^{*}$ of $t-IFS\;A_{t}$ of the universe U is defined as:

$$A_{t}^{*}=\left\{a_{1}\in U:\mu_{A_{t}}\left(a_{1}\right)>0\;\mathrm{and}\;\nu_{A_{t}}\left(a_{1}\right)<1\right\}$$

The functions $\mu_{A_{t}}\left(a_{1}\right)$ and $\nu_{A_{t}}\left(a_{1}\right)$ respectively assign the membership and non-membership degrees to the element $a_{1}$ in $A_{t}$.

Definition 5 [41] The level set of $t-IFS\;A_{t}$ may be defined as:

$$\Lambda \left(A_{t}\right)=\left\{\left(\rho ,\eta \right):\mu_{A_{t}}\left(a_{1}\right)=\rho ,\;\nu_{A_{t}}\left(a_{1}\right)=\eta ,\;\mathrm{for}\;\mathrm{some}\;a_{1}\in U\right\}.$$

$\mu_{A_{t}}\left(a_{1}\right)$ and $\nu_{A_{t}}\left(a_{1}\right)$ stand for the degrees of membership and non-membership of the element $a_{1}$ in $A_{t}$, respectively.

Definition 6 [41] Let $A_{t}$ be a t−IFSG of group G. The subgroup ${ℭ}_{\left(\rho ,\eta \right)}\left(A_{t}\right)\;\rho ,\eta \in \left[0,1\right]$ with 0≤ρ+η≤1 is called a level subgroup of $A_{t}$

The symbol ${ℭ}_{\left(\rho ,\eta \right)}\left(A_{t}\right)$ represent the (ρ,η)− cut set of t−IFSG $A_{t}$.

Definition 7 [40] A $t-IFSG\;A_{t}$ is called t-intuitionistic fuzzy normal subgroup (t−IFNSG) of G if it satisfies the following conditions:

1) $\mu_{A_{t}}\left(a_{1}b_{1}\right)=\mu_{A_{t}}\left(b_{1}a_{1}\right)$
2) $\nu_{A_{t}}\left(a_{1}b_{1}\right)=\nu_{A_{t}}\left(b_{1}a_{1}\right),\forall a_{1},b_{1}\in G$.

The terms $\mu_{A_{t}}\left(a_{1}b_{1}\right)$ and $\nu_{A_{t}}\left(a_{1}b_{1}\right)$ respectively denote the membership and non-membership degrees of the element $\prime \prime a_{1}b_{1}\prime \prime$ in $A_{t}\text{.}$ Likewise, the terminologies $\mu_{A_{t}}\left(b_{1}a_{1}\right)$ and $\nu_{A_{t}}\left(b_{1}a_{1}\right)$ respectively characterize the membership and non-membership degrees of the element $\prime \prime b_{1}a_{1}\prime \prime$ in $A_{t}$.

Theorem 1 [41] A $t-IFS\;A_{t}$ of a group G is t-IFSG if and only if its each level set is a subgroup of group G.

Theorem 2 [41] A $t-IFS\;A_{t}$ of a group G is t-IFSG of G iff $C_{\left(\rho ,\eta \right)}\left(A_{t}\right)$ is a normal subgroup of G where ρ and η are positive real numbers such that their sum lies in the closed unit interval.

Theorem 3 [41] Consider a $t-IFSG\;A_{t}$ of a group G and

$$\Lambda \left(A_{t}\right)=\left\{\left(\rho ,\eta \right):\mu_{A_{t}}\left(a_{1}\right)=\rho ,\nu_{A_{t}}\left(a_{1}\right)=\eta \;\mathrm{for}\;\mathrm{some}\;a_{1}\in U\right\}\text{.}$$

Then the family of level subgroups ${I_{A}}_{t}=\left\{C_{\left(\rho_{i},\eta_{i}\right)}\left(A_{t}\right):0\leq i\leq r\right\}$ constitutes all the level subgroups of $A_{t}$.

Definition 8 [41] Consider a t-IFSG $A_{t}$ of a finite group G and $b_{1}\in G$. Then the t-intuitionistic fuzzy order of the element $b_{1}\in A_{t}$ is denoted by $t-{IFO}_{A_{t}}\left(b_{1}\right)$ and is defined as:

$$t-{IFO}_{A_{t}}\left(b_{1}\right)=\left|H\left(b_{1}\right)\right|$$

Where $ℍ\left(b_{1}\right)=\left\{a_{1}\in G:\mu_{A_{t}}\left(a_{1}\right)\geq \mu_{A_{t}}\left(b_{1}\right),\nu_{A_{t}}\left(a_{1}\right)\leq \nu_{A_{t}}\left(b_{1}\right)\right\}.$

The set $H\left(b_{1}\right)$ is calculated by the elements ${{\prime \prime b}_{1}}^{\prime \prime }$ in G. The set $H\left(b_{1}\right)$ comprises of elements of “$a_{1}$” that belong to the group G and have a membership degree $\mu_{A_{t}}$ in $A_{t}$ greater than or equal to that element “$b_{1}$” and a non-membership degree $\nu_{A_{t}}$ in $A_{t}$ less than that element “$b_{1}$”. Here, $\mu_{A_{t}}\left(a_{1}\right),\mu_{A_{t}}\left(b_{1}\right)$ and $\nu_{A_{t}}\left(a_{1}\right),\nu_{A_{t}}\left(b_{1}\right)$, respectively, represents the membership and non-membership degrees of elements $a_{1}$ and $b_{1}$ in $A_{t}$.

Theorem 4 [42] Any subgroup H of a group G can be visualized as a level subgroup of some t-IFSG of G.

Theorem 5 [42] If $t-{IFO}_{A_{t}}\left(a_{1}\right)=n$ then $t-{IFO}_{A_{t}}\left(a_{1}^{m}\right)=\frac{t-{IFO}_{A_{t}}\left(a_{1}\right)}{\left(n,m\right)}$, for some integer m.

Theorem 6 [42] Let G be a finite group and $A_{t}$ be a t-IFSG of G. Then $\left[G:A_{t}\right]$ divides O(G).

## 3 Conjugacy class of t-intuitionistic fuzzy subgroup of a group

In this section, we define the notion of a t-intuitionistic fuzzy conjugate element of a finite group G and establish the classification of these newly defined elements. We also discuss the class equation of the t−IFSG of the finite group G.

Definition 9 Let $A_{t}$ be a t-IFSG of a finite group G and $a_{1},b_{1}\in G\text{.}$ Then $a_{1}$ is t-intuitionistic fuzzy conjugate (t-IFC) to $b_{1}$ (written as $a_{1}{∼}_{A_{t}}b_{1})$ if there exists a non-identity element $g_{1}\in G$ such that $\mu_{A_{t}}\left(a_{1}\right)=\mu_{A_{t}}\left(g_{1}^{-1}b_{1}g_{1}\right)$ and $\nu_{A_{t}}\left(a_{1}\right)=\nu_{A_{t}}\left(g_{1}^{-1}b_{1}g_{1}\right)\text{.}$

Example 1 The dihedral group $D_{3}$ of degree 3 is defined as:

$$D_{3}=\left\langle r,s:r^{3}=s^{2}=1,sr=r^{2}s\right\rangle \text{.}$$

The t-IFSG $A_{t}$ of $D_{3}$ for t=0.70 is defined as follows: $\mu_{A_{0.70}}\left(z_{1}\right)=\left\{ \begin{array}{cc} \begin{array}{c} 0.70\\ 0.50\\ 0.40 \end{array} & \begin{array}{c} if\;z_{1}=1\\ if\;z_{1}=s\\ if\;z_{1}\in \left\{r,r^{2},rs,r^{2}s\right\} \end{array} \end{array}\right.$and $\nu_{0.70}\left(z_{1}\right)=\left\{ \begin{array}{cc} \begin{array}{c} 0.30\\ 0.40\\ 0.50 \end{array} & \begin{array}{c} if\;z_{1}=1\\ if\;z_{1}=s\\ if\;z_{1}\in \left\{r,r^{2},rs,r^{2}s\right\} \end{array} \end{array}\right.$.

In the light of definition (9), we obtain that $s{∼}_{A_{0.70}}s\text{,}$ $r{∼}_{A_{0.70}}r^{2}$ and $rs{∼}_{A_{0.70}}r^{2}s$.

Theorem 7 If $a_{1}$ is t-IFC to $b_{1}$ then $t-{IFO}_{A_{t}}\left(a_{1}\right)=t-{IFO}_{A_{t}}\left(b_{1}\right)\text{,}$ where $a_{1},b_{1}\in G\text{.}$

**Proof.** Since $a_{1}$ and $b_{1}$ are t-IFC then there exists $g_{1}\in G$ such that

$\mu_{A_{t}}\left(a_{1}\right)=\mu_{A_{t}}\left(g_{1}^{-1}b_{1}g_{1}\right)$ and $\nu_{A_{t}}\left(a_{1}\right)=\nu_{A_{t}}\left(g_{1}^{-1}b_{1}g_{1}\right)$.

Consider

$$\mu_{A_{t}}\left(a_{1}^{2}\right)=\mu_{A_{t}}\left(g_{1}^{-1}b_{1}g_{1}\cdot g_{1}^{-1}b_{1}g_{1}\right)=\mu_{A_{t}}\left(g_{1}^{-1}b_{1}^{2}g_{1}\right)$$

and

$$\nu_{A_{t}}\left(a_{1}^{2}\right)=\nu_{A_{t}}\left(g_{1}^{-1}b_{1}g_{1}\cdot g_{1}^{-1}b_{1}g_{1}\right)=\nu_{A_{t}}\left(g_{1}^{-1}b_{1}^{2}g_{1}\right)\text{.}$$

The application of the method of induction in the above relation gives us:

$$\mu_{A_{t}}\left(a_{1}^{n}\right)=\mu_{A_{t}}\left(g_{1}^{-1}b_{1}^{n}g_{1}\right),\nu_{A_{t}}\left(a_{1}^{n}\right)=\nu_{A_{t}}\left(g_{1}^{-1}b_{1}^{n}g_{1}\right)\text{,}$$

and

$$\mu_{A_{t}}\left(b_{1}^{n}\right)=\mu_{A_{t}}\left(g_{1}^{-1}a_{1}^{n}g_{1}\right),\nu_{A_{t}}\left(b_{1}^{n}\right)=\nu_{A_{t}}\left(g_{1}^{-1}a_{1}^{n}g_{1}\right)\text{.}$$

Consider $t-{IFO}_{A_{t}}\left(a_{1}\right)=m_{1}$ and $t-{IFO}_{A_{t}}\left(b_{1}\right)=m_{2}$. This implies that

$$\mu_{A_{t}}\left(b_{1}^{m_{1}}\right)=\mu_{A_{t}}\left(g_{1}^{-1}a_{1}^{m_{1}}g_{1}\right)=\mu_{A_{t}}\left(e\right).$$

In view of Theorem (5), we have

(1) $$m_{1}|m_{2}\text{.}$$

Moreover, $\mu_{A_{t}}\left(a_{1}^{m_{2}}\right)=\mu_{A_{t}}\left(g_{1}^{-1}b_{1}^{m_{2}}g_{1}\right)=\mu_{A_{t}}\left(e\right)$.

The application of Theorem (5) gives that

(2) $$m_{2}|m_{1}\text{.}$$

From relations (1) and (2), we have the required result.

Definition 10 Let $A_{t}$ be a t-IFSG of a finite group G. The t-intuitionistic fuzzy conjugacy class (t-IFℂℂl) of an element $a_{1}\in G$ is defined as:

$$Cl_{A_{t}}\left(a_{1}\right)=\left\{b_{1}\in G:b_{1}{∼}_{A_{t}}a_{1}\right\}=\left\{b_{1}\in G:\mu_{A_{t}}\left(b_{1}\right)=\mu_{A_{t}}\left(g_{1}^{-1}a_{1}g_{1}\right)\;\mathrm{and}\;\nu_{A_{t}}\left(b_{1}\right)=\nu_{A_{t}}\left(g_{1}^{-1}a_{1}g_{1}\right),g_{1}\in G\right\}.$$

Example 2 The finite presentation of the dihedral group $D_{4}$ of degree 4 is defined as: $D_{4}=\left\langle r,s:r^{4}=s^{2}=1,sr=r^{3}s\right\rangle$.

The t-IFSG $A_{t}$ of $D_{4}$ correspond to the value t=0.70 is given as: $\mu_{A_{0.70}}\left(z_{1}\right)=\left\{ \begin{array}{cc} \begin{array}{c} 0.70\\ 0.65\\ 0.60\\ 0.50 \end{array} & \begin{array}{c} if\;z_{1}=1\\ if\;z_{1}=s\\ if\;z_{1}\in \left\{r^{2},r^{2}s\right\}\\ if\;z_{1}\in \left\{r,r^{3},rs,r^{3}s\right\} \end{array} \end{array}\right.$ and $\nu_{A_{0.70}}\left(z_{1}\right)=\left\{ \begin{array}{cc} \begin{array}{c} 0.30\\ 0.35\\ 0.40\\ 0.50 \end{array} & \begin{array}{c} if\;z_{1}=1\\ if\;z_{1}=s\\ if\;z_{1}\in \left\{r^{2},r^{2}s\right\}\\ if\;z_{1}\in \left\{r,r^{3},rs,r^{3}s\right\} \end{array} \end{array}\right.$.

The application of definition (10) yields that $Cl_{A_{0.70}}\left(1\right)=\left\{1\right\}$, $Cl_{A_{0.70}}\left(r\right)=\left\{r,r^{3}\right\}$, $Cl_{A_{0.70}}\left(r^{2}\right)=\left\{r^{2}\right\}$, $Cl_{A_{0.70}}\left(s\right)=\left\{s\right\}$, $Cl_{A_{0.70}}\left(r^{2}s\right)=\left\{r^{2}s\right\}$ and $Cl_{A_{0.70}}\left(rs\right)=\left\{{rs,r}^{3}s\right\}$.

The following theorem describes that the t-IFC relation between elements of t-IFSG in a group is an equivalence relation.

Proposition 1 The t-intuitionistic fuzzy conjugacy between elements $t-IFSG\;A_{t}\;$ of a group G is an equivalence relation.

**Proof. Reflexivity:** The application of the definition (9) to any element $a_{1}\in G\text{,}$ we have $\mu_{A_{t}}\left(a_{1}\right)=\mu_{A_{t}}\left(e^{-1}a_{1}e\right)$ and $\nu_{A_{t}}\left(a_{1}\right)=\nu_{A_{t}}\left(e^{-1}a_{1}e\right)\text{,}$ where e∈G.

It follows that $a_{1}{∼}_{A_{t}}a_{1}$.

**Symmetry:** Consider $a_{1}{∼}_{A_{t}}b_{1}$ so that there exists an element $g_{1}\in G$ such that $\mu_{A_{t}}\left(a_{1}\right)=\mu_{A_{t}}\left(g_{1}^{-1}b_{1}g_{1}\right)$ and $\nu_{A_{t}}\left(a_{1}\right)=\nu_{A_{t}}\left(g_{1}^{-1}b_{1}g_{1}\right),a_{1},b_{1}\in G$.

This implies that $\mu_{A_{t}}\left(g_{1}a_{1}g_{1}^{-1}\right)=\mu_{A_{t}}\left(g_{1}g_{1}^{-1}b_{1}g_{1}g_{1}^{-1}\right)$ and $\nu_{A_{t}}\left(g_{1}a_{1}g_{1}^{-1}\right)=\nu_{A_{t}}\left(g_{1}g_{1}^{-1}b_{1}g_{1}g_{1}^{-1}\right)$.

This further implies that $\mu_{A_{t}}\left(g_{1}^{-1}a_{1}g_{1}\right)=\mu_{A_{t}}\left(b_{1}\right)$ and $\nu_{A_{t}}\left(g_{1}^{-1}a_{1}g_{1}\right)=\nu_{A_{t}}\left(b_{1}\right)$

This shows that $b_{1}{∼}_{A_{t}}a_{1}$.

**Transitivity:** For any $a_{1},b_{1},c_{1}\in G$. Consider $a_{1}{∼}_{A_{t}}b_{1}$ and $b_{1}{∼}_{A_{t}}c_{1}$ there exist two elements $g_{1},g_{2}\in G$ such that

$$\mu_{A_{t}}\left(a_{1}\right)=\mu_{A_{t}}\left(g_{1}^{-1}b_{1}g_{1}\right),\nu_{A_{t}}\left(a_{1}\right)=\nu_{A_{t}}\left(g_{1}^{-1}b_{1}g_{1}\right)$$

and $\mu_{A_{t}}\left(b_{1}\right)=\mu_{A_{t}}\left(g_{2}^{-1}c_{1}g_{2}\right)\text{,}$ $\nu_{A_{t}}\left(b_{1}\right)=\nu_{A_{t}}\left(g_{2}^{-1}c_{1}g_{2}\right)$ where $a_{1},b_{1},c_{1}\in G$.

This implies that $\mu_{A_{t}}\left(a_{1}\right)=\mu_{A_{t}}\left(g_{1}^{-1}{g_{2}^{-1}c}_{1}g_{2}g_{1}\right)$ and $\nu_{A_{t}}\left(a_{1}\right)=\nu_{A_{t}}\left(g_{1}^{-1}{g_{2}^{-1}c}_{1}g_{2}g_{1}\right)$

This shows that $\mu_{A_{t}}\left(a_{1}\right)=\mu_{A_{t}}\left({\left(g_{2}g_{1}\right)}^{-1}c_{1}\;\left(g_{2}g_{1}\right)\right)$ and $\nu_{A_{t}}\left(a_{1}\right)=\nu_{A_{t}}\left({\left(g_{2}g_{1}\right)}^{-1}c_{1}\;\left(g_{2}g_{1}\right)\right)$

This means that $a_{1}{∼}_{A_{t}}c_{1}\text{.}$ Consequently, this proves the equivalence properties of the t-intuitionistic fuzzy conjugacy relation.

Remark 1

1) In view of the above theorem, the group G is partitioned into equivalence classes, such classes are called t-IFℂℂl of the group G. It is important to note that one can obtain a different partition of G corresponding to different t-IFSG defined on it. Whereas there is only one partition of group G in classical group theory. The significance of this approach of getting many partitions of a group G is to obtain an economic solution of a decision-making problem under consideration.
2) The t-IFℂℂl of an element of a finite abelian group G is always a singleton set.

The following result indicates the conditions under which the t-IFℂℂl of two elements are equal.

Theorem 8 The t-IFℂℂl of the elements $a_{1}$ and $b_{1}$ are equal if and only if $a_{1}$ and $b_{1}$ are t-IFℂ to each other.

**Proof.** Suppose that $a_{1}$ and $b_{1}$ are t-IFC to each other. Consider $g_{1}\in Cl_{A_{t}}\left(a_{1}\right)\text{,}$ then by using definition (10), we have $g_{1}{∼}_{A_{t}}a_{1}\text{.}$ Since $g_{1}{∼}_{A_{t}}a_{1}$ and $a_{1}{∼}_{A_{t}}b_{1}$. By the transitive property, we have $g_{1}{∼}_{A_{t}}b_{1}\text{.}$ Thus $g_{1}\in Cl_{A_{t}}\left(b_{1}\right)\text{.}$ This shows that $Cl_{A_{t}}\left(a_{1}\right)\subseteq Cl_{A_{t}}\left(b_{1}\right)\text{.}$ In the same way, we obtain $Cl_{A_{t}}\left(b_{1}\right)\subseteq Cl_{A_{t}}\left(a_{1}\right)\text{.}$ Consequently, $Cl_{A_{t}}\left(a_{1}\right)=Cl_{A_{t}}\left(b_{1}\right)$.

Conversely, let $Cl_{A_{t}}\left(a_{1}\right)=Cl_{A_{t}}\left(b_{1}\right)\text{.}$ This implies that $g_{1}{∼}_{A_{t}}a_{1}$ and $g_{1}{∼}_{A_{t}}b_{1},g_{1}\in G$. Consequently, $a_{1}{∼}_{A_{t}}b_{1}$.

Definition 11 If $A_{t}$ is a t-IFSG of a finite group G, the centralizer $Ć\left(A_{t}\right)$ of $A_{t}$ in G is defined as: $Ć\left(A_{t}\right)=\left\{a_{1}\in G:\mu_{A_{t}}\left(a_{1}g_{1}\right)=\mu_{A_{t}}\left(g_{1}a_{1}\right)\;\mathrm{and}\;\nu_{A_{t}}\left(a_{1}g_{1}\right)=\nu_{A_{t}}\left(g_{1}a_{1}\right),\forall g_{1}\in G\right\}$

Lemma 1 Let $A_{t}$ be a t-IFSG of a group G and $T=\left\{a_{1}\in G:\mu_{A_{t}}\left(a_{1}g_{1}a_{1}^{-1}g_{1}^{-1}\right)=\mu_{A_{t}}\left(e\right)\;\mathrm{and}\;\nu_{A_{t}}\left(a_{1}g_{1}a_{1}^{-1}g_{1}^{-1}\right)=\nu_{A_{t}}\left(e\right),\forall g_{1}\in G\right\}$ then $T=Ć\left(A_{t}\right)$.

**Proof.** Let $a_{1}\in T$ then for all $g_{1}\in G\text{,}$ we get

$$\mu_{A_{t}}\left(a_{1}g_{1}{\left(g_{1}a_{1}\right)}^{-1}\right)=\mu_{A_{t}}\left(a_{1}g_{1}g_{1}^{-1}a_{1}^{-1}\right)=\mu_{A_{t}}\left(e\right)$$

and

$$\nu_{A_{t}}\left(a_{1}g_{1}{\left(g_{1}a_{1}\right)}^{-1}\right)=\nu_{A_{t}}\left(a_{1}g_{1}g_{1}^{-1}a_{1}^{-1}\right)=\nu_{A_{t}}\left(e\right)\text{.}$$

This implies that $\mu_{A_{t}}\left(a_{1}g_{1}\right)=\mu_{A_{t}}\left(g_{1}a_{1}\right)$ and$\nu_{A_{t}}\left(g_{1}a_{1}\right)=\nu_{A_{t}}\left(a_{1}g_{1}\right),\forall g_{1}\in G\text{.}$ Thus $T\subseteq Ć\left(A_{t}\right)$.

Furthermore, if $a_{1}\in Ć\left(A_{t}\right)$ then $\mu_{A_{t}}\left(a_{1}g_{1}\right)=\mu_{A_{t}}\left(g_{1}a_{1}\right)\;\mathrm{and}\;\nu_{A_{t}}\left(g_{1}a_{1}\right)=\nu_{A_{t}}\left(a_{1}g_{1}\right)$.

This implies that $\mu_{A_{t}}\left(a_{1}g_{1}a_{1}^{-1}g_{1}^{-1}\right)=\mu_{A_{t}}\left(e\right)$ and $\nu_{A_{t}}\left(a_{1}g_{1}a_{1}^{-1}g_{1}^{-1}\right)=\nu_{A_{t}}\left(e\right),\forall a_{1},g_{1}\in G$.

So, $a_{1}\in T\text{.}$ Thus $T⊇Ć\left(A_{t}\right)\text{.}$ Hence $T=Ć\left(A_{t}\right)$.

Definition 12 If $A_{t}$ is a t-IFSG of a finite group G, the centralizer of an element of $A_{t}$ in G (written as ${Ĉ}_{A_{t}}\left(a_{1}\right))$ is defined as: ${Ĉ}_{A_{t}}\left(a_{1}\right)=\left\{g_{1}\in G:\mu_{A_{t}}\left(a_{1}g_{1}a_{1}^{-1}g_{1}^{-1}\right)=\mu_{A_{t}}\left(e\right)\;\mathrm{and}\;\nu_{A_{t}}\left(a_{1}g_{1}a_{1}^{-1}g_{1}^{-1}\right)=\nu_{A_{t}}\left(e\right)\right\}$.

Example 3 Consider $0.80-IFSG\;A_{0.80}$ of dihedral group $D_{3}$ as follows: $\mu_{A_{0,80}}\left(z_{1}\right)=\left\{ \begin{array}{cc} 0.80 & if\;z_{1}\in \left\{{1,r,r}^{2}\right\}\\ 0.50 & if\;z_{1}\in \left\{s,r^{2}s,rs\right\} \end{array}\right.$ and $\nu_{A_{0.20}}\left(z_{1}\right)=\left\{ \begin{array}{cc} 0.20 & if\;z_{1}\in \left\{{1,r,r}^{2}\right\}\\ 0.45 & if\;z_{1}\in \left\{s,r^{2}s,rs\right\} \end{array}\right.$.

In the light of definitions (11) and (12), we acquire:

$$Ć\left(A_{0.80}\right)=D_{3}\text{.}$$

$${Ĉ}_{A_{0.80}}\left(1\right)=D_{3}\text{,}$$

$${Ĉ}_{A_{0.80}}\left(r\right)={Ĉ}_{A_{0.80}}\left(r^{2}\right)=\left\{1,r,r^{2}\right\}$$

and

$${Ĉ}_{A_{0.80}}\left(s\right)={Ĉ}_{A_{0.80}}\left(rs\right)={Ĉ}_{A_{0.80}}\left(r^{2}s\right)=D_{3}$$

Theorem 9 The t-intuitionistic fuzzy centralizer of $A_{t}$ is a subgroup of G.

**Proof.** For any two elements $g_{1},g_{2}\in Ć\left(A_{t}\right)$, we have

(3) $$A_{t}\left(g_{1}a_{1}g_{1}^{-1}a_{1}^{-1}\right)=A_{t}\left(e\right),\forall a_{1}\in G$$

(4) $$A_{t}\left(g_{2}b_{1}g_{2}^{-1}b_{1}^{-1}\right)=A_{t}\left(e\right),\forall b_{1}\in G$$

Consider

$$A_{t}\left(\left(g_{1}g_{2}\right)c_{1}{\left(g_{1}g_{2}\right)}^{-1}c_{1}^{-1}\right)=A_{t}\left(g_{1}g_{2}c_{1}g_{2}^{-1}g_{1}^{-1}c_{1}^{-1}\right)$$

$$=A_{t}\left(g_{1}g_{2}c_{1}g_{2}^{-1}c_{1}^{-1}c_{1}g_{1}^{-1}c_{1}^{-1}\right)$$

$$=A_{t}\left(g_{1}c_{1}g_{1}^{-1}c_{1}^{-1}\right)$$

$$=A_{t}\left(e\right)\text{.}$$

This shows that $g_{1}g_{2}\in Ć\left(A_{t}\right)$.

Moreover, consider $A_{t}\left(g_{1}^{-1}a_{1}g_{1}a_{1}^{-1}\right)$.

Substituting $a_{1}=g_{1}d_{1}$ in the above relation, we get

$$A_{t}\left(g_{1}^{-1}a_{1}g_{1}a_{1}^{-1}\right)=A_{t}\left(g_{1}^{-1}g_{1}d_{1}g_{1}d_{1}^{-1}g_{1}^{-1}\right)$$

$$=A_{t}\left(d_{1}g_{1}d_{1}^{-1}g_{1}^{-1}\right)$$

$$=A_{t}\left(e\right)$$

Thus, $g_{1}^{-1}\in Ć\left(A_{t}\right)$.

Consequently, $Ć\left(A_{t}\right)$ is a subgroup of G.

Lemma 2 Let $A_{t}\;be\;a\;t-IFSG$ of a finite group G. Then $a_{1}\in Ć\left(A_{t}\right)$ if

$$\mu_{A_{t}}\left(a_{1}g_{1}g_{2}\ldots g_{s}\right)=\mu_{A_{t}}\left(g_{1}a_{1}g_{2}\ldots g_{s}\right)=\ldots \mu_{A_{t}}\left(g_{1}g_{2}\ldots g_{s}a_{1}\right)$$

and

$$\nu_{A_{t}}\left(a_{1}g_{1}g_{2}\ldots g_{s}\right)=\nu_{A_{t}}\left(g_{1}a_{1}g_{2}\ldots g_{s}\right)=\ldots \nu_{A_{t}}\left(g_{1}g_{2}\ldots g_{s}a_{1}\right),\forall g_{1},g_{2},\ldots g_{s}\in G.$$

**Proof.** We prove by induction to s. Suppose $a_{1}\in C\left(A_{t}\right)\text{.}$ Then

$$\mu_{A_{t}}\left({a_{1}g}_{1}g_{2}\right)=\mu_{A_{t}}\left(g_{1}g_{2}a_{1}\right),\forall g_{1},g_{2}\in G\text{.}$$

Assume that $\mu_{A_{t}}\left(a_{1}g_{1}g_{2}\ldots g_{s}\right)=\mu_{A_{t}}\left(g_{1}a_{1}g_{2}\ldots g_{s}\right)=\ldots \mu_{A_{t}}\left(g_{1}g_{2}\ldots g_{s}a_{1}\right)$.

Then

$$\mu_{A_{t}}\left(a_{1}g_{1}g_{2}\ldots \left(g_{s}g_{s+1}\right)\right)=\mu_{A_{t}}\left(g_{1}a_{1}g_{2}\ldots \left(g_{s}g_{s+1}\right)\right)$$

⋮

$$=\mu_{A_{t}}\left(g_{1}g_{2}\ldots a_{1}\left(g_{s}g_{s+1}\right)\right)$$

$$=\mu_{A_{t}}\left(g_{1}g_{2}\ldots \left(g_{s}g_{s+1}\right)a_{1}\right)$$

and

$$\mu_{A_{t}}\left(a_{1}\left(g_{1}g_{2}\right)\ldots g_{s}g_{s+1}\right)=\mu_{A_{t}}\left(\left(g_{1}g_{2}\right)a_{1}\ldots g_{s}g_{s+1}\right)$$

⋮

$$=\mu_{A_{t}}\left(\left(g_{1}g_{2}\right)\ldots g_{s}a_{1}g_{s+1}\right)$$

$$=\mu_{A_{t}}\left(\left(g_{1}g_{2}\right)a_{1}\ldots .g_{s}g_{s+1}a_{1}\right),\forall g_{1},g_{2},\ldots g_{s}\in G$$

Now, we obtain the required result by applying the similar arguments for non-membership function $\nu_{A_{t}}$.

This completes the proof.

Corollary 1 Let $A_{t}$ be a t-IFSG of a group G, then

1) If $A_{t}$ is a t-IFℕSG of a group G then $Ć\left(A_{t}\right)⊴G$.
2) If $A_{t}$ is a t-IFSG of an abelian group G then $Ć\left(A_{t}\right)=G$.

Theorem 10 Let $A_{t}$ be a t-IFℕSG of a finite group G then

$$\left|Cl_{A_{t}}\left(a_{1}\right)\right|=\frac{\left|G\right|}{\left|{Ĉ}_{A_{t}}\left(a_{1}\right)\right|},a_{1}\in G\text{.}$$

**Proof.** Consider the collection H of all disjoint cosets of ${Ĉ}_{A_{t}}\left(a_{1}\right)$ in G is given by:

$$ℍ=\left\{g_{1}{Ĉ}_{A_{t}}\left(a_{1}\right),g_{2}{Ĉ}_{A_{t}}\left(a_{1}\right),g_{3}{Ĉ}_{A_{t}}\left(a_{1}\right),\ldots ,g_{s}{Ĉ}_{A_{t}}\left(a_{1}\right):g_{s}\in G\right\}$$

Moreover, the left decomposition of G as a disjoint union of cosets of ${Ĉ}_{A_{t}}\left(a_{1}\right)$ in G is given by: $G=\overset{s}{\underset{i=1}{\cup }}g_{i}{Ĉ}_{A_{t}}\left(a_{1}\right)$, $g_{i}\in G$.

It follows that $O\left(G\right)=s\cdot O\left({Ĉ}_{A_{t}}\left(a_{1}\right)\right)\text{.}$ Define a mapping $\varphi :H\to Cl_{A_{t}}\left(a_{1}\right)$ by

$$\varphi \left(g_{1}{Ĉ}_{A_{t}}\left(a_{1}\right)\right)=A_{t}\left(b_{1}^{-1}a_{1}b_{1}\right)\text{.}$$

As φ is well defined because for any two elements $b_{1},c_{1}\in G\text{,}$ we have $b_{1}{Ĉ}_{A_{t}}\left(a_{1}\right)=c_{1}{Ĉ}_{A_{t}}\left(a_{1}\right)$ this implies that $b_{1}^{-1}c_{1}\in {Ĉ}_{A_{t}}\left(a_{1}\right)$.

By using definition (12), we have

$$A_{t}\left(a_{1}\left(b_{1}^{-1}c_{1}\right)a_{1}^{-1}{\left(b_{1}^{-1}c_{1}\right)}^{-1}\right)=A_{t}\left(e\right)$$

This shows that $A_{t}\left(b_{1}^{-1}a_{1}b_{1}\right)=A_{t}\left(c_{1}^{-1}a_{1}c_{1}\right)$. Consequently, $\varphi \left(b_{1}C_{A_{t}}\left(a_{1}\right)\right)=\varphi \left(c_{1}C_{A_{t}}\left(a_{1}\right)\right)$.

As φ is injective as for any two elements $b_{1},c_{1}\in G$, we have $\varphi \left(b_{1}C_{A_{t}}\left(a_{1}\right)\right)=\varphi \left(c_{1}C_{A_{t}}\left(a_{1}\right)\right)$.

This implies that $A_{t}\left(b_{1}^{-1}a_{1}b_{1}\right)=A_{t}\left(c_{1}^{-1}a_{1}c_{1}\right)\text{.}$ This further implies that

$$A_{t}\left(a_{1}\left(b_{1}^{-1}c_{1}\right)a_{1}^{-1}{\left(b_{1}^{-1}c_{1}\right)}^{-1}\right)=A_{t}\left(e\right)$$

This means that $b_{1}^{-1}c_{1}\in {Ĉ}_{A_{t}}\left(a_{1}\right)\text{.}$ Thus $b_{1}{Ĉ}_{A_{t}}\left(a_{1}\right)=c_{1}{Ĉ}_{A_{t}}\left(a_{1}\right)$.

Moreover, one can easily prove that φ is surjective.

Thus, there is a one-to-one correspondence between H and $Cl_{A_{t}}\left(a_{1}\right)$.Hence $O\left(H\right)=O\left(Cl_{A_{t}}\left(a_{1}\right)\right)\text{.}$ Consequently, $\left|Cl_{A_{t}}\left(a_{1}\right)\right|=\frac{\left|G\right|}{\left|{Ĉ}_{A_{t}}\left(a_{1}\right)\right|}$.

Corollary 2 The cardinality of the t-IFℂℂl of an element of the finite group G divides the order of G.

**Proof.** Since ${Ĉ}_{A_{t}}\left(a_{1}\right)$ is a subgroup of G, its order and index divide the order of G by Lagrange’s Theorem. However, the index of ${Ĉ}_{A_{t}}\left(a_{1}\right)$ is equal to the number of elements in $Cl_{A_{t}}\left(a_{1}\right)$ which therefore divides the order of G.

Definition 13 If $A_{t}$ is a t-IFSG of a finite group G, the t-intuitionistic fuzzy normalizer $N\left(A_{t}\right)$ of $A_{t}$ in G is defined as:

$$N\left(A_{t}\right)=\left\{g_{1}\in G:\mu_{A_{t}}\left(a_{1}\right)=\mu_{A_{t}}\left(g_{1}^{-1}a_{1}g_{1}\right)\;\mathrm{and}\;\nu_{A_{t}}\left(a_{1}\right)=\nu_{A_{t}}\left(g_{1}^{-1}a_{1}g_{1}\right),\forall a_{1}\in G\right\}\text{.}$$

Example 4 Consider the t-IFSG $A_{t}$ of dihedral group $D_{3}$ for the value t=0.70 as follows: $\mu_{A_{0,70}}\left(z_{1}\right)=\left\{ \begin{array}{cc} 0.70 & if\;z_{1}\in \left\{{1,r,r}^{2}\right\}\\ 0.40 & if\;z_{1}\in \left\{s,r^{2}s,rs\right\} \end{array}\right.$ and $\nu_{A_{0.70}}\left(z_{1}\right)=\left\{ \begin{array}{cc} 0.30 & if\;z_{1}\in \left\{{1,r,r}^{2}\right\}\\ 0.25 & if\;z_{1}\in \left\{s,r^{2}s,rs\right\} \end{array}\right.$.

With reference to definition (13), we obtain $N\left(A_{0.70}\right)=\left\{1,r,r^{2}\right\}$

Corollary 3 Let $A_{t}$ be a t-IFSG of a group G then:

1) If $A_{t}$ is a t-IFSG of a group G then $N\left(A_{t}\right)\leq G$.
2) If $A_{t}$ is a t-IFNSG of a group G then $N\left(A_{t}\right)=G$.
3) $Ć\left(A_{t}\right)$ is a normal subgroup of $N\left(A_{t}\right)$.

Definition 14 The class equation of t-IFSG $A_{t}$ of the finite group G is defined as:

$$\left|G\right|=\sum_{a_{1}\in G}\left|Cl_{A_{t}}\left(a_{1}\right)\right|$$

where $Cl_{A_{t}}\left(a_{1}\right)$ is the t-IFCCl of an element of $A_{t}$ of G.

Definition 15 The Class equation of t-IFNSG $A_{t}$ of the finite group G is defined as**:**

$$\left|G\right|=\sum_{a_{1}\in G}\left|\left[G:{Ĉ}_{A_{t}}\left(a_{1}\right)\right]\right|$$

The following examples demonstrate the aforementioned algebraic facts.

Example 5 The class equation of $0.70-IFSG\;A_{0.70}$ of $D_{4}$ is $\left|D_{4}\right|=1+2+1+1+1+2=8$ (See example 2).

Example 6 Consider the $0.70-IFNSG\;A_{0.70}$ of dihedral group $D_{10}$ as follows:

$$\mu_{A_{0.70}}\left(z_{1}\right)=\left\{ \begin{array}{cc} \begin{array}{c} 0.70\\ 0.65\\ 0.45 \end{array} & \begin{array}{c} if\;z_{1}=1\\ if\;z_{1}\in \left\{r,r^{2},r^{3},r^{4}\right\}\\ if\;z_{1}\in \left\{s,rs,r^{2}s,r^{3}s,r^{4}s\right\} \end{array} \end{array}\right.$$

and

$$\nu_{A_{0.70}}\left(z_{1}\right)=\left\{ \begin{array}{cc} \begin{array}{c} 0.30\\ 0.35\\ 0.50 \end{array} & \begin{array}{c} if\;z_{1}=1\\ if\;z_{1}\in \left\{r,r^{2},r^{3},r^{4}\right\}\\ if\;z_{1}\in \left\{s,rs,r^{2}s,r^{3}s,r^{4}s\right\} \end{array} \end{array}\right.$$

In accordance with the definition (12), we have

$${Ĉ}_{A_{0.70}}\left(1\right)=D_{10},{Ĉ}_{A_{0.70}}\left(r\right)=\left\{1,r,r^{2},r^{3},r^{4}\right\},{Ĉ}_{A_{0.70}}\left(s\right)=\left\{1,s\right\},{Ĉ}_{A_{0.70}}\left(rs\right)=\left\{1,rs\right\}\text{,}$$

${Ĉ}_{A_{0.70}}\left(r^{2}s\right)=\left\{1,r^{2}s\right\},{Ĉ}_{A_{0.70}}\left(r^{3}s\right)=\left\{1,r^{3}s\right\}\text{,}$ and ${Ĉ}_{A_{0.70}}\left(r^{4}s\right)=\left\{1,r^{4}s\right\}$.

The class equation of $0.70-IFNSG\;A_{0.70}$ of $D_{10}$ is

|G|=5+2+2+1=10.

## 4 Algebraic characteristics of t-intuitionistic fuzzy p− subgroup

In this section, we initiate the idea of the t-intuitionistic fuzzy p− subgroup of t-IFSG and establish the various algebraic properties of this phenomenon. Furthermore, we prove the t-intuitionistic fuzzy version of the Cauchy Theorem.

Definition 16 A t-IFSG $A_{t}$ of a group, G is a t-intuitionistic fuzzy p-subgroup if $t-{IFO}_{A_{t}}\left(a_{1}\right)$ is a power of prime $p,\forall a_{1}\in G\text{.}$

Theorem 11 [42]. Let $A_{t}$ be a t-IFNSG of a finite group G, then the set $G_{t}=\left\{a_{1}\in G:\mu_{A_{t}}\left(a_{1}\right)=\mu_{A_{t}}\left(e\right)\;\mathrm{and}\;\nu_{A_{t}}\left(a_{1}\right)=\nu_{A_{t}}\left(e\right)\right\}$ is a normal subgroup of G.

In the following result, we establish a condition under which a t-IFSG is a t-intuitionistic fuzzy p- subgroup.

Theorem 12 Consider a t-IFSG $A_{t}$ of a finite group G such that

$$G_{t}=\left\{a_{1}\in G:\mu_{A_{t}}\left(a_{1}\right)=\mu_{A_{t}}\left(e\right)\;\mathrm{and}\;\nu_{A_{t}}\left(a_{1}\right)=\nu_{A_{t}}\left(e\right)\right\}$$

is a normal subgroup of G then $A_{t}$ is a t-intuitionistic fuzzy p- subgroup if and only if $G/G_{t}$ is a p− group.

**Proof.** In view of definition (16) and for any element $a_{1}$ in G, we have $t-{IFO}_{A_{t}}\left(a_{1}\right)=p^{s}$ for some non-negative integer s and so $a_{1}^{p^{s}}\in G_{t}\text{.}$ Thus $G/G_{t}$ is a p− group. Conversely, let $G/G_{t}$ is a p− group. If $a_{1}\in G$ then $a_{1}^{p^{s}}\in G_{t}$ for some nonnegative integer s and so $A_{t}\left(a_{1}^{p^{s}}\right)=A_{t}\left(e\right)\text{.}$ Consequently, $A_{t}$ is a t-intuitionistic fuzzy p-subgroup.

Theorem 13 If $t-{IFO}_{A_{t}}\left(a_{1}\right)=m_{1}n_{1}$ for some coprime positive integer $m_{1}$ and $n_{1}$, then there exist $b_{1},b_{2}\in G$ such that $a_{1}=b_{1}b_{2}=b_{2}b_{1},t-{IFO}_{A_{t}}\left(b_{1}\right)=m_{1}$ and $t-{IFO}_{A_{t}}\left(b_{2}\right)=n_{1}\text{.}$

**Proof.** Assume that $t-{IFO}_{A_{t}}\left(a_{1}\right)=m_{1}n_{1}$. Since $\left(m_{1},n_{1}\right)=1$ then there exist integers $p_{1}$ and $q_{1}$ such that $m_{1}p_{1}+n_{1}q_{1}=1$. Here $\left(m_{1},q_{1}\right)=\left(n_{1},p_{1}\right)=1\text{.}$ Let $b_{1}=a_{1}^{n_{1}p_{1}}$ and $b_{1}=a_{1}^{m_{1}q_{1}}$ then $a_{1}=b_{1}b_{2}=b_{2}b_{1}$.

By using Theorem (5), we have

$$t-{IFO}_{A_{t}}\left(b_{1}\right)=t-{IFO}_{A_{t}}\left(a_{1}^{n_{1}p_{1}}\right)=m_{1}$$

and

$$t-{IFO}_{A_{t}}\left(b_{2}\right)=t-{IFO}_{A_{t}}\left(a_{1}^{m_{1}q_{1}}\right)=n_{1}\text{.}$$

Theorem 14 **(t-Intuitionistic Fuzzification of Cauchy Theorem)**. Let $A_{t}$ be a t-IFSG of a finite group G and $t-IFO\left(A_{t}\right)=p_{1}^{r}m_{1}\text{,}$ where p is prime and $\left(p_{1},m_{1}\right)=1$, then there is an element $a_{1}\in G$ such that $t-{IFO}_{A_{t}}\left(a_{1}\right)=p_{1}^{s}$, for each nonnegative integer s≤r.

**Proof.** Since $t-IFO\left(A_{t}\right)$ is the greatest common divisor of $t-{IFO}_{A_{t}}\left(a_{1}\right)$, where $a_{1}\in G$ there is an element $a_{1}$ in G such that $t-{IFO}_{A_{t}}\left(a_{1}\right)=p_{1}^{s}\text{.}$ By using the induction method on s and the Cauchy Theorem in classical group theory we have an element $a_{1}\in G$ such that $t-{IFO}_{A_{t}}\left(a_{1}\right)=p_{1}^{s}$.

Corollary 4 If $A_{t}$ is a t-IFSG of an abelian group G and $t-IFO\left(A_{t}\right)=m_{1}n_{1}$ for some $m_{1},n_{1}\in Z\text{,}$ then there is an element $a_{1}$ in G such that $t-{IFO}_{A_{t}}\left(a_{1}\right)=m_{1}\text{.}$

Remark 2 Let $A_{t}$ be a t-IFSG of a group G satisfying the following conditions for some prime $p_{1}$ then $H_{p_{1}}=\left\{a_{1}\in G:\left(t-{IFO}_{A_{t}}\left(a_{1}\right),p_{1}\right)=1\right\}$ and $L_{p_{1}}=\left\{a_{1}\in G:t-{IFO}_{A_{t}}\left(a_{1}^{p_{1}}\right)\right\}$ are constitute a subgroup of G.

Theorem 15 Let $A_{t}$ be any t-IFSG of a group G such that the t-intuitionistic fuzzy index of $A_{t}$ is p, where p is the smallest prime p divisor the order of G then $A_{t}$ is a t-IFNSG of G.

**Proof**. Define a subgroup H of index p as follows:

$$H=\left\{z_{1}\in G:A_{t}\left(z_{1}\right)=A_{t}\left(e\right)\right\}\text{.}$$

Then the group G acts on the left cosets $\frac{G}{H}$ by left multiplication as:

$$\left\{a_{i}H:1\leq i\leq p_{1}\right\}$$

Now, consider the permutation representation of G on the cosets of H given by the map

$$\rho :G\to \rho_{a}$$

where

$$\rho_{a}:a_{i}H\to aa_{i}H,1\leq i\leq p_{1}\text{.}$$

As is well known, ρ is a homomorphism of G into the symmetric group. Further, the kernel of the map ρ is the core of H. By the First Isomorphism Theorem of groups, the quotient group G/Core(H) is isomorphic to the subgroup of the symmetric group, thus O(G/Core(H)) divides $p_{1}!$ by Lagrange’s Theorem. Since O(G/H) is p, it follows that the O(H/Core(H)) divides $\left(p_{1}-1\right)!\text{.}$ Now since the O(H) divides the O(G), we obtain H=Core(H); otherwise, we get a contradiction to the fact that $p_{1}$ is the smallest prime divisor the O(G). As Core(H) is always a normal subgroup, it follows that H is a normal subgroup of G. Moreover, G/H is abelian. It follows that $a_{1}b_{1}=b_{1}a_{1}h_{1}$ for some $h_{1}\in H$ implies that

$$\mu_{A_{t}}\left(a_{1}b_{1}\right)=\mu_{A_{t}}\left(b_{1}a_{1}h_{1}\right)$$

In view of definition (2), we have

$$\geq min\left\{\mu_{A_{t}}\left(b_{1}a_{1}\right),\mu_{A_{t}}\left(h_{1}\right)\right\}$$

$$=min\left\{\mu_{A_{t}}\left(b_{1}a_{1}\right),\mu_{A_{t}}\left(e\right)\right\}$$

Consequently, $\mu_{A_{t}}\left(a_{1}b_{1}\right)\geq \mu_{A_{t}}\left(b_{1}a_{1}\right)$.

Similarly, $\mu_{A_{t}}\left(a_{1}b_{1}\right)\leq \mu_{A_{t}}\left(b_{1}a_{1}\right)$.

Thus $\mu_{A_{t}}\left(a_{1}b_{1}\right)=\mu_{A_{t}}\left(b_{1}a_{1}\right),\forall a_{1},b_{1}\in G$.

The same procedure is applied to get $\nu_{A_{t}}\left(a_{1}b_{1}\right)=\nu_{A_{t}}\left(b_{1}a_{1}\right),\forall a_{1},b_{1}\in G$.

This concludes that $A_{t}$ is t-IFNSG of G.

Corollary 5 Let $A_{t}$ be any t-IFSG of a group G such that the t-intuitionistic fuzzy index of $A_{t}$ is 2, then $A_{t}$ is t-IFNSG of G.

Definition 17 Let $A_{t}$ be any t-IFSG of a finite group G and

$$ℍ=\left\{a_{1}\in G:\mu_{A_{t}}\left(a_{1}\right)=\mu_{A_{t}}\left(e\right)\;\mathrm{and}\;\nu_{A_{t}}\left(a_{1}\right)=\nu_{A_{t}}\left(e\right)\right\}$$

Then $A_{t}$ is t-intuitionistic fuzzy abelian if H is an abelian subgroup of G.

Theorem 16 A t-IFSG $A_{t}$ is t-intuitionistic fuzzy abelian if $t-IFO\left(A_{t}\right)=p_{1}^{2}\text{,}$ where $p_{1}$ a is prime.

**Proof.** The result is obtained by a simple application of the definition (16).

## 5 t-Intuitionistic fuzzy conjugate subgroup of t-intuitionistic fuzzy subgroups

In this section, we define the notions of equivalent t-IFSG and t-intuitionistic fuzzy conjugate subgroup of t-IFSG. We also establish the various algebraic aspects of this phenomenon. Moreover, we establish the relation under which a t-IFSG is a t-intuitionistic fuzzy conjugate subgroup.

Theorem 17 If $A_{t}$ is a t-IFSG of a finite group G and $\left(\rho_{i},\eta_{i}\right),\left(\rho_{j},\eta_{j}\right)\in \Lambda \left(A_{t}\right)$ such that $C_{\left(\rho_{i},\eta_{i}\right)}\left(A_{t}\right)=C_{\left(\rho_{j},\eta_{j}\right)}\left(A_{t}\right)$, then $\left(\rho_{i},\eta_{i}\right)=\left(\rho_{j},\eta_{j}\right)$.

**Proof.** Let $a_{i}\in C_{\left(\rho_{i},\eta_{i}\right)}\left(A_{t}\right)$ and $a_{j}\in C_{\left(\rho_{j},\eta_{j}\right)}\left(A_{t}\right)$. As $a_{i}\in C_{\left(\rho_{i},\eta_{i}\right)}\left(A_{t}\right)$, we have

$$A_{t}\left(a_{i}\right)=\left(\rho_{i},\eta_{i}\right)\geq \left(\rho_{j},\eta_{j}\right)$$

Similarly, $\left(\rho_{j},\eta_{j}\right)\geq \left(\rho_{i},\eta_{i}\right)$. Consequently, $\left(\rho_{i},\eta_{i}\right)=\left(\rho_{j},\eta_{j}\right)$.

Theorem 18 Let $A_{t}$ and $B_{t}$ be two t-IFSG of a finite group G having an identical family of level subgroups. If

$$\Lambda \left(A_{t}\right)=\left\{\left(\rho_{0},\eta_{0}\right),\left(\rho_{1},\eta_{1}\right),\ldots ,\left(\rho_{r},\eta_{r}\right)\right\}$$

and

$$\Lambda \left(B_{t}\right)=\left\{\left(\rho_{0}^{'},\eta_{0}^{'}\right),\left(\rho_{1}^{'},\eta_{1}^{'}\right),\ldots ,\left(\rho_{s}^{'},\eta_{s}^{'}\right)\right\}$$

where $1=\rho_{0}\geq \rho_{1}\geq \ldots \geq \rho_{r},0=\eta_{0}\leq \eta_{1}\leq \ldots \leq \eta_{r}$ and $1=\rho_{0}^{\prime }\geq \rho_{1}^{\prime }\geq \ldots \geq \rho_{s}^{\prime }\text{,}$ $0=\eta_{0}^{\prime }\leq \eta_{1}^{\prime }\leq \ldots \leq \eta_{s}^{\prime }$ then

1) r=s
2) $C_{\left(\rho_{i},\eta_{i}\right)}\left(A_{t}\right)=C_{\left(\rho_{i},\eta_{i}\right)}\left(B_{t}\right),0\leq i\leq r$
3) For any element $a_{1}\in G$ such that $A_{t}\left(a_{1}\right)=\left(\rho_{i},\eta_{i}\right)$ then $B_{t}\left(a_{1}\right)=\left(\rho_{i}^{\prime },\eta_{i}^{\prime }\right),0\leq i\leq r$.

**Proof. (1)** By using Theorem (3), the only level subgroups of $A_{t}$ and $B_{t}$ have the same families

$$I_{A_{t}}=I_{B_{t}}\text{.}$$

As $A_{t}$ and $B_{t}$ have the same family of level subgroups, it follows that r=s.

**(2)** There are two chains of level subgroups, which can be identified using part (1) of the theorem (18) and the theorem (3)

$$C_{\left(\rho_{0},\;\eta_{0}\right)}\left(A_{t}\right)\subseteq C_{\left(\rho_{1},\;\eta_{1}\right)}\left(A_{t}\right)\subseteq ...\subseteq C_{\left(\rho_{r},\;\eta_{r}\right)}\left(A_{t}\right)=G$$

and

$$C_{\left(\rho_{0}^{'},\;\eta_{0}^{'}\right)}\left(B_{t}\right)\subseteq C_{\left(\rho_{1}^{'},\;\eta_{1}^{'}\right)}\left(B_{t}\right)\subseteq ...\subseteq C_{\left(\rho_{s}^{'},\;\eta_{s}^{'}\right)}\left(B_{t}\right)=G$$

From this, it now follows that if: $\left(\rho_{i},\eta_{i}\right),\left(\rho_{j},\eta_{j}\right)\in \Lambda \left(A_{t}\right)$ such that $\left(\rho_{i},\eta_{i}\right)>\left(\rho_{j},\eta_{j}\right)\text{,}$ then

(5) $$C_{\left(\rho_{i},\;\eta_{i}\right)}\left(A_{t}\right)\subseteq C_{\left(\rho_{j},\;\eta_{j}\right)}\left(A_{t}\right)$$

If $\left(\rho_{i}^{'},\eta_{i}^{'}\right),\left(\rho_{j}^{'},\eta_{j}^{'}\right)\in \Lambda \left(B_{t}\right)$ such that $\left(\rho_{i}^{\prime },\eta_{i}^{\prime }\right)>\left(\rho_{j}^{\prime },\eta_{j}^{\prime }\right)$.

then

(6) $$C_{\left(\rho_{i}^{\prime },\eta_{i}^{\prime }\right)}\left(B_{t}\right)\subseteq C_{\left(\rho_{j}^{\prime },\eta_{j}^{\prime }\right)}\left(B_{t}\right)$$

Since the two families of level subgroups are identical, it is clear that $C_{\left(\rho_{0},\eta_{0}\right)}\left(A_{t}\right)=C_{\left(\rho_{0}^{\prime },\eta_{0}^{\prime }\right)}\left(B_{t}\right)$.

Now by hypothesis $C_{\left(\rho_{1},\eta_{1}\right)}\left(A_{t}\right)=C_{\left(\rho_{j}^{\prime },\eta_{j}^{\prime }\right)}\left(B_{t}\right)$ for some j>0. Suppose that $C_{\left(\rho_{1},\eta_{1}\right)}\left(A_{t}\right)=C_{\left(\rho_{j}^{\prime },\eta_{j}^{\prime }\right)}\left(B_{t}\right)$ for some j>1. Again, we have that $C_{\left(\rho_{i},\eta_{i}\right)}\left(A_{t}\right)=C_{\left(\rho_{1}^{\prime },\eta_{1}^{\prime }\right)}\left(B_{t}\right)$ for some ($\rho_{i},\eta_{i})<\left(\rho_{1},\eta_{1}\right)\text{.}$ It is clear that $\left(\rho_{i},\eta_{i}\right)\neq \left(\rho_{1},\eta_{1}\right)$.

According to relation (5), we obtain

(7) $$C_{\left(\rho_{i},\eta_{i}\right)}\left(A_{t}\right)<C_{\left(\rho_{j}^{\prime },\eta_{j}^{\prime }\right)}\left(B_{t}\right)\text{.}$$

The application of relation (6) yields that

(8) $$C_{\left(\rho_{j}^{\prime },\eta_{j}^{\prime }\right)}\left(B_{t}\right)<C_{\left(\rho_{i},\eta_{i}\right)}\left(A_{t}\right)\text{.}$$

Relations (7) and (8) allow us to derive that

$$C_{\left(\rho_{1},\eta_{1}\right)}\left(A_{t}\right)=C_{\left(\rho_{1}^{\prime },\eta_{1}^{\prime }\right)}\left(B_{t}\right)\text{.}$$

By using the induction method on i, we obtain

$$C_{\left(\rho_{i},\eta_{i}\right)}\left(A_{t}\right)=C_{\left(\rho_{i}^{\prime },\eta_{i}^{\prime }\right)}\left(B_{t}\right),0\leq i\leq r\text{.}$$

**(3)** Consider any non-zero element $a_{1}\in G$ such that $A_{t}\left(a_{1}\;\right)=\left(\rho_{i},\eta_{i}\right)$ and $B_{t}\left(a_{1}\right)=\left(\rho_{j}^{\prime },\eta_{j}^{\prime }\right)$.

By using part (2) of Theorem (18), we have $C_{\left(\rho_{i},\eta_{i}\right)}\left(A_{t}\right)=C_{\left(\rho_{i}^{\prime },\eta_{i}^{\prime }\right)}\left(B_{t}\right)\text{.}$ So, $a_{1}\in C_{\left(\rho_{i}^{\prime },\eta_{i}^{\prime }\right)}\left(B_{t}\right)$ implies that $B_{t}\left(a_{1}\right)=\left(\rho_{j}^{\prime },\eta_{j}^{\prime }\right)$ such that $\left(\rho_{j}^{\prime },\eta_{j}^{\prime }\right)\geq \left(\rho_{i}^{\prime },\eta_{i}^{\prime }\right)$.

By applying relation (8), we get

$$C_{\left(\rho_{j}^{\prime },\eta_{j}^{\prime }\right)}\left(B_{t}\right)\leq C_{\left(\rho_{i}^{\prime },\eta_{i}^{\prime }\right)}\left(B_{t}\right)\text{.}$$

Again, by using part (2) of Theorem (18), we have

$$C_{\left(\rho_{j},\eta_{j}\right)}\left(A_{t}\right)=C_{\left(\rho_{j}^{\prime },\eta_{j}^{\prime }\right)}\left(B_{t}\right)\text{.}$$

So, as $a_{1}\in C_{\left(\rho_{j}^{\prime },\eta_{j}^{\prime }\right)}\left(B_{t}\right)\text{,}$ we have that $a_{1}\in C_{\left(\rho_{j},\eta_{j}\right)}\left(A_{t}\right)$ and so $A_{t}\left(a_{1}\right)=\left(\rho_{i},\eta_{i}\right)\geq \left(\rho_{j},\eta_{j}\right)\text{.}$.

Now, from relation (5), we get

$$C_{\left(\rho_{i},\eta_{i}\right)}\left(A_{t}\right)\leq C_{\left(\rho_{j},\eta_{j}\right)}\left(A_{t}\right)\text{.}$$

But by using part (2) of Theorem (18), we have $C_{\left(\rho_{i},\eta_{i}\right)}\left(A_{t}\right)=C_{\left(\rho_{i}^{\prime },\eta_{i}^{\prime }\right)}\left(B_{t}\right)$ and $C_{\left(\rho_{j},\eta_{j}\right)}\left(A_{t}\right)=C_{\left(\rho_{j}^{\prime },\eta_{j}^{\prime }\right)}\left(B_{t}\right)$.

Consequently, we have that $C_{\left(\rho_{i}^{\prime },\eta_{i}^{\prime }\right)}\left(B_{t}\right)\leq C_{\left(\rho_{j}^{\prime },\eta_{j}^{\prime }\right)}\left(B_{t}\right)$ which contradicts the fact $C_{\left(\rho_{j}^{\prime },\eta_{j}^{\prime }\right)}\left(B_{t}\right)\leq C_{\left(\rho_{i}^{\prime },\eta_{i}^{\prime }\right)}\left(B_{t}\right)$ obtained earlier unless we have that $C_{\left(\rho_{j}^{\prime },\eta_{j}^{\prime }\right)}\left(B_{t}\right)=C_{\left(\rho_{i}^{\prime },\eta_{i}^{\prime }\right)}\left(B_{t}\right)\text{.}$.

Using Theorem (17), it follows that $\left(\rho_{j}^{\prime },\eta_{j}^{\prime }\right)=\left(\rho_{i}^{\prime },\eta_{i}^{\prime }\right)$.

Theorem 19 Let $A_{t}$ and $B_{t}$ be any two t-IFSG of a finite group G. Then $A_{t}=B_{t}$ if and only if $\Lambda \left(A_{t}\right)=\Lambda \left(B_{t}\right)\text{.}$

**Proof.** If $A_{t}=B_{t}$ then obviously $\Lambda \left(A_{t}\right)=\Lambda \left(B_{t}\right)\text{.}$ Conversely, assume that $\Lambda \left(A_{t}\right)=\Lambda \left(B_{t}\right)\text{.}$ For convenience, let us denote $\Lambda \left(A_{t}\right)=\left\{\left(\rho_{0},\eta_{0}\right),\left(\rho_{1},\eta_{1}\right),...,\left(\rho_{r},\eta_{r}\right)\right\}$ where $1=\rho_{0}\geq \rho_{1}\geq ...\geq \rho_{r}$, $0=\eta_{0}\leq \eta_{1}\leq ...\leq \eta_{r}$ and $\Lambda \left(B_{t}\right)=\left\{\left(\rho_{0}^{'},\eta_{0}^{'}\right),\left(\rho_{1}^{'},\eta_{1}^{'}\right),...,\left(\rho_{r}^{'},\eta_{r}^{'}\right)\right\}$ where $1=\rho_{0}^{\prime }\geq \rho_{1}^{\prime }\geq ...\geq \rho_{r}^{\prime }$, $0=\eta_{0}^{\prime }\leq \eta_{1}^{\prime }\leq ...\leq \eta_{r}^{\prime }\text{.}$ Therefore $\left(\rho_{k_{0}},\eta_{k_{0}}\right)=\left(\rho_{s}^{\prime },\eta_{s}^{\prime }\right)$ for some $k_{0}\text{.}$ Suppose if possible $\left(\rho_{k_{0}},\eta_{k_{0}}\right)\neq \left(\rho_{0},\eta_{0}\right)\text{.}$ So, $\left(\rho_{k_{0}},\eta_{k0}\right)<\left(\rho_{1},\eta_{1}\right)\text{.}$ Now, $\left(\rho_{1}^{'},\eta_{1}^{'}\right)\in \Lambda \left(B_{t}\right)$ and so $\left(\rho_{1}^{\prime },\eta_{1}^{\prime }\right)=\left(\rho_{k_{1}},\eta_{k_{1}}\right)$ for some $k_{1}\text{.}$ We have $\left(\rho_{0}^{\prime },\eta_{0}^{\prime }\right)>\left(\rho_{1}^{\prime },\eta_{1}^{\prime }\right)$ and $\left(\rho_{k_{0}},\eta_{k_{0}}\right)>\left(\rho_{k_{1}},\eta_{k_{1}}\right)\text{.}$ Proceeding in this way, we have

(9) $$\left(\rho_{k_{0}},\eta_{k_{0}}\right)>\left(\rho_{k_{1}},\eta_{k_{1}}\right)>...>\left(\rho_{k_{r}},\eta_{k_{r}}\right)$$

where

(10) $$\left(\rho_{0}^{\prime },\eta_{0}^{\prime }\right)=\left(\rho_{k_{0}},\eta_{k_{0}}\right)>\left(\rho_{0},\eta_{0}\right)\text{.}$$

However, relations (9) and (10) contradict the fact that

$$\Lambda \left(A_{t}\right)=\Lambda \left(B_{t}\right)\text{.}$$

Hence, we have $\left(\rho_{0},\eta_{0}\right)=\left(\rho_{0}^{\prime },\eta_{0}^{\prime }\right)\text{.}$ Arranging in this manner, we obtain that $\left(\rho_{i},\eta_{i}\right)=\left(\rho_{i}^{\prime },\eta_{i}^{\prime }\right),0\leq i\leq r\text{.}$ Now, let $b_{0},b_{1},...,b_{r}$ be distinct elements of G such that $A_{t}\left(b_{i}\right)=\left(\rho_{i},\eta_{i}\right),0\leq i\leq r$.

Then by Theorem (18), we have that

$$B_{t}\left(b_{i}\right)=\left(\rho_{i}^{\prime },\eta_{i}^{\prime }\right),0\leq i\leq r\text{.}$$

Since $\left(\rho_{i},\eta_{i}\right)=\left(\rho_{i}^{\prime },\eta_{i}^{\prime }\right)\text{,}$ it follows that $A_{t}\left(b\right)=B_{t}\left(b\right),\forall b\in G\text{.}$ Hence, $A_{t}=B_{t}$.

Definition 18 Let $A_{t}$ and $B_{t}$ be any two t-IFSG of a group G then $A_{t}$ and $B_{t}$ are said to be equivalent (denoted by $A_{t}↔B_{t})\text{,}$ if $I_{A_{t}}=I_{B_{t}}$.

Remark 3 Let ℱ denotes the collection of t-IFSG of a finite group G. Define a relation on ℱ as follows: $A_{t}↔B_{t}$ if and only if $A_{t}$ and $B_{t}$ have the same family of level subgroups for any $A_{t}$ and $B_{t}$ in ℱ.

Lemma 3 The relation ‘↔’ is an equivalence relation.

**Proof.** Let $\left[A_{t}\right]$ denotes the equivalence class containing $A_{t}$, where $A_{t}\in F$. As group G is finite, the number of possible distinct level subgroups of G is finite since each level subgroup is a subgroup of group G. From Theorem (3), it follows that the number of possible chains of level subgroups is also finite. As each equivalence class is characterized completely by its chain of level subgroups.

Definition 19 Let $A_{t}$ and $B_{t}$ be any two t-IFSG of a group G. We say that $A_{t}$ is t-intuitionistic fuzzy conjugate (t-IFCSG) to $B_{t}$ if there exists $a_{1}\in G$ such that $\mu_{A_{t}}\left(b_{1}\right)=\mu_{B_{t}}\left(a_{1}^{-1}b_{1}a_{1}\right)$ and $\nu_{A_{t}}\left(b_{1}\right)=\nu_{B_{t}}\left(a_{1}^{-1}b_{1}a_{1}\right),\forall b_{1}\in G$.

It is interesting to note that the relation of conjugacy between t-IFSG is an equivalence. Consequently, the family of t-IFSG of a group G is a union of pairwise disjoint classes of t-IFSG each consisting of equivalent t-IFSG.

Theorem 20 Let $A_{t}$ be a t-IFSG and $B_{t}$ be a t-IFS of a group G. If $A_{t}$ and $B_{t}$ are t-IFCSG of G then $B_{t}$ is a t-IFSG of group G.

**Proof.** Since $A_{t}$ and $B_{t}$ are t-IFCSG of G. In view of the definition (18) and for some $a_{1}\in G$, we have $\mu_{B_{t}}\left(b_{1}\right)=\mu_{A_{t}}\left(a_{1}^{-1}b_{1}a_{1}\right)$ and $\nu_{B_{t}}\left(b_{1}\right)=\nu_{A_{t}}\left(a_{1}^{-1}b_{1}a_{1}\right),\forall b_{1}\in G$.

Consider

$$\mu_{B_{t}}\left(b_{1}\right)=\mu_{B_{t}}\left(e_{1}b_{1}e_{1}\right)$$

$$=\mu_{B_{t}}\left(a_{1}^{-1}a_{1}b_{1}a_{1}^{-1}a_{1}\right)$$

$$=\mu_{A_{t}}\left(a_{1}^{-1}b_{1}a_{1}\right)\text{.}$$

Consider

$$\mu_{B_{t}}\left({a_{1}b}_{1}\right)=\mu_{B_{t}}\left(e_{1}a_{1}e_{1}b_{1}e_{1}\right)$$

$$=\mu_{B_{t}}\left(c_{1}^{-1}c_{1}a_{1}c_{1}^{-1}c_{1}b_{1}c_{1}^{-1}c_{1}\right)$$

$$=\mu_{A_{t}}\left(c_{1}a_{1}c_{1}^{-1}c_{1}b_{1}c_{1}^{-1}\right)$$

Since $A_{t}$ is a t-IFSG of the group G.

$$\geq \min\left\{\mu_{A_{t}}\left(c_{1}a_{1}c_{1}^{-1}\right),\mu_{A_{t}}\left(c_{1}b_{1}c_{1}^{-1}\right)\right\}$$

$$=\min\left\{\mu_{B_{t}}\left(a_{1}\right),\mu_{B_{t}}\left(b_{1}\right)\right\}$$

Consequently, $\mu_{B_{t}}\left(a_{1}b_{1}\right)\geq \min\left\{\mu_{B_{t}}\left(a_{1}\right),\mu_{B_{t}}\left(b_{1}\right)\right\}$.

Also $\mu_{B_{t}}\left(b_{1}^{-1}\right)=\mu_{A_{t}}\left(a_{1}b_{1}^{-1}a_{1}^{-1}\right)$.

$$=\mu_{A_{t}}\left(a_{1}b_{1}a_{1}^{-1}\right)$$

$$=\mu_{B_{t}}\left(b_{1}\right)\text{.}$$

The same procedure is applied for $\nu_{B_{t}}$.

This shows that $B_{t}$ is a t-IFSG of group G.

Theorem 21 Let $A_{t}$ and $B_{t}$ be any two t−IFSG of a group G then $A_{t}$ and $B_{t}$ are t-IFCSG of G if and only if $A_{t}=B_{t}\text{.}$

**Proof.** Assume that $A_{t}$ and $B_{t}$ are t-IFCSG of a group G. With reference to definition (18), we have $\mu_{B_{t}}\left(b_{1}\right)=\mu_{A_{t}}\left(a_{1}^{-1}b_{1}a_{1}\right)$ and $\nu_{B_{t}}\left(b_{1}\right)=\nu_{A_{t}}\left(a_{1}^{-1}b_{1}a_{1}\right),\forall b_{1}\in G$.

Consider $a_{1}b_{1}=b_{1}a_{1}\in G$ then $\mu_{A_{t}}\left(a_{1}b_{1}\right)=\mu_{B_{t}}\left(a_{1}^{-1}b_{1}a_{1}a_{1}\right)$. This implies that

$$\mu_{A_{t}}\left(a_{1}b_{1}\right)=\mu_{B_{t}}\left(b_{1}a_{1}\right)\text{.}$$

For some $a_{1}=e_{1}\in G\text{,}$ we have $\mu_{A_{t}}\left(e_{1}b_{1}\right)=\mu_{B_{t}}\left(b_{1}e_{1}\right)\text{.}$ This further implies that

$$\mu_{A_{t}}\left(b_{1}\right)=\mu_{B_{t}}\left(b_{1}\right)\text{.}$$

Consequently $\mu_{A_{t}}=\mu_{B_{t}}\text{.}$ Similarly, $\nu_{A_{t}}=\nu_{B_{t}}\text{.}$ This shows that $A_{t}=B_{t}$.

Conversely, suppose that $A_{t}=B_{t}\text{.}$ Let $\mu_{A_{t}}=\mu_{B_{t}}\text{.}$ This implies that $\mu_{A_{t}}\left(b_{1}\right)=\mu_{B_{t}}\left(b_{1}\right)$.

This means that $\mu_{A_{t}}\left(b_{1}\right)=\mu_{B_{t}}\left(e_{1}^{-1}b_{1}e_{1}\right),\forall b_{1},e_{1}\in G$.

In the same way, we have $\nu_{A_{t}}\left(b_{1}\right)=\nu_{B_{t}}\left(e_{1}^{-1}b_{1}e_{1}\right),\forall b_{1},e_{1}\in G$.

Consequently, $A_{t}$ and $B_{t}$ are t-IFCSG of G.

Corollary 6 If $A_{t}$ and $B_{t}$ are any two t-IFCSG of a group G then

$$t-IFO\left(A_{t}\right)=t-IFO\left(B_{t}\right)\text{.}$$

Theorem 22 Let $A_{t}$ be any t-IFSG of a group G. For any element $g_{1}$ in G define a map $A_{t}^{g_{1}}:G\to \left[0,1\right]$ by $\mu_{A_{t}^{g_{1}}}\left(a_{1}\right)=\mu_{A_{t}}\left(g_{1}^{-1}a_{1}g_{1}\right)$ and $\nu_{A_{t}^{g_{1}}}\left(a_{1}\right)=\nu_{A_{t}}\left(g_{1}^{-1}a_{1}g_{1}\right),\forall a_{1}\in G$. Then the following statements are true:

1) $A_{t}^{g_{1}}$ is a t-IFSG and $A_{t}^{g_{1}}$ is a t-IFCSG of G determined by $A_{t}$ and $g_{1}$ in G.
2) If $\left|\Lambda \left(A_{t}\right)\right|=2$, then $A_{t}^{g_{1}}=\left\{g_{1}C_{\left(\rho_{0},\eta_{0}\right)}\left(A_{t}\right)g_{1}^{-1},G\right\}$, where $\rho_{0}=\mu_{A_{t}}\left(e\right)$ and $\eta_{0}=\nu_{A_{t}}\left(e\right)$.

**Proof. (1).** Define a map $I_{g_{1}}:G\to G$ by $I_{g_{1}}\left(a_{1}\right)=g_{1}^{-1}a_{1}g_{1},\forall a_{1}\in G\text{.}$ Then $I_{g_{1}}\in AutG$ and $A_{t}^{g_{1}}=A_{t}\circ I_{g_{1}}=I_{g_{1}}^{-1}\left(A_{t}\right)$. Since the inverse image of a t-IFSG by a group homomorphism is always a t-IFSG, it follows that $A_{t}^{g_{1}}$ is a t-IFSG. Thus $A_{t}^{g_{1}}$ is a t-IFCSG of group G determined by $A_{t}$ and $g_{1}$ in G is trivial.

**(2).** Since $A_{t}^{g_{1}}=I_{g_{1}}^{-1}\left(A_{t}\right)$, it follows that $\Lambda \left(A_{t}^{g_{1}}\right)=\Lambda \left(A_{t}\right)\text{.}$ Let $\Lambda \left(A_{t}\right)=\left\{\left(\rho_{i},\eta_{i}\right):0\leq i\leq 1\right\}$In view of definition (3), we have

$$b_{1}\in C_{\left(\rho_{i},\eta_{i}\right)}\left(A_{t}^{g_{1}}\right)$$

$\Leftrightarrow \mu_{A_{t}^{g_{1}}}\left(b_{1}\right)\geq \rho_{i}$ and $\nu_{A_{t}^{g_{1}}}\left(b_{1}\right)\leq \eta_{i}$.

$\Leftrightarrow \mu_{A_{t}}\left(g_{1}^{-1}b_{1}g_{1}\right)\geq \rho_{i}\;and\;\nu_{A_{t}}\left(g_{1}^{-1}b_{1}g_{1}\right)\leq \eta_{i}$.

$$\Leftrightarrow g_{1}^{-1}b_{1}g_{1}\in C_{\left(\rho_{i},\eta_{i}\right)}\left(A_{t}\right)$$

$$\Leftrightarrow b_{1}\in g_{1}C_{\left(\rho_{i},\eta_{i}\right)}\left(A_{t}\right)g_{1}^{-1}$$

Thus $C_{\left(\rho_{i},\eta_{i}\right)}\left(A_{t}^{g_{1}}\right)=g_{1}C_{\left(\rho_{i},\eta_{i}\right)}\left(A_{t}\right)g_{1}^{-1}$.

Consequently, we obtain that

$$I_{A_{t}^{g_{1}}}=\left\{C_{\left(\rho_{0},\eta_{0}\right)}\left(A_{t}^{g_{1}}\right),C_{\left(\rho_{1},\eta_{1}\right)}\left(A_{t}^{g_{1}}\right)\right\}=\left\{g_{1}C_{\left(\rho_{0},\eta_{0}\right)}\left(A_{t}\right)g_{1}^{-1},G\right\}\text{.}$$

Theorem 23 If the chain of the level subgroups of $t-IFSG\;A_{t}$ is given by:

$$C_{\left(\rho_{0},\eta_{0}\right)}\left(A_{t}\right)\subseteq C_{\left(\rho_{1},\eta_{1}\right)}\left(A_{t}\right)\subseteq \ldots \subseteq C_{\left(\rho_{r},\eta_{r}\right)}\left(A_{t}\right)=G$$

then the chain of level subgroups $t-IFCSG\;A_{t}^{g_{1}}$, the t-IFCSG $A_{t}^{g_{1}}$ of G is determined by $A_{t}$ and $g_{1}\in G$ is given by

$$g_{1}C_{\left(\rho_{0},\eta_{0}\right)}\left(A_{t}\right)g_{1}^{-1}\subseteq g_{1}C_{\left(\rho_{1},\eta_{1}\right)}\left(A_{t}\right)g_{1}^{-1}\subseteq \ldots \subseteq g_{1}C_{\left(\rho_{r},\eta_{r}\right)}\left(A_{t}\right)g_{1}^{-1}=G\text{.}$$

**Proof.** In view of Theorem (22), we have $C_{\left(\rho_{i},\eta_{i}\right)}\left(A_{t}^{g_{1}}\right)=g_{1}C_{\left(\rho_{i},\eta_{i}\right)}\left(A_{t}\right)g_{1}^{-1}$.

From this, we conclude that the chain of level subgroups of $A_{t}^{g_{1}}$ is given by

$$g_{1}{ℭ}_{\left(\rho_{0},\eta_{0}\right)}\left(A_{t}\right)g_{1}^{-1}\subseteq g_{1}{ℭ}_{\left(\rho_{1},\eta_{1}\right)}\left(A_{t}\right)g_{1}^{-1}\subseteq ...\subseteq g_{1}{ℭ}_{\left(\rho_{r},\eta_{r}\right)}\left(A_{t}\right)g_{1}^{-1}=G$$

In the following result, we develop a mechanism to obtain a t-IFCSG corresponding to a t-IFSG in the framework of the concept of level subgroups.

Theorem 24 If the chain of the level subgroups of t-IFSG $A_{t}$ is given by

$$C_{\left(\rho_{0},\eta_{0}\right)}\left(A_{t}\right)\subseteq C_{\left(\rho_{1},\eta_{1}\right)}\left(A_{t}\right)\subseteq ...\subseteq C_{\left(\rho_{r},\eta_{r}\right)}\left(A_{t}\right)=G$$

then there exists a t-IFSG $B_{t}$ of G whose chain of level subgroups is given by

$${g_{1}C}_{\left(\rho_{0},\eta_{0}\right)}\left(B_{t}\right)g_{1}^{-1}\subseteq g_{1}C_{\left(\rho_{1},\eta_{1}\right)}\left(B_{t}\right)g_{1}^{-1}\subseteq \ldots \subseteq g_{1}C_{\left(\rho_{r},\eta_{r}\right)}\left(B_{t}\right)g_{1}^{-1}=G$$

where $g_{1}\in G$ and $B_{t}=A_{t}^{g_{1}}$.

**Proof.** Consider the $t-IFS\;B_{t}$ of the group G as follows:

$$\mu_{B_{t}}\left(a_{1}\right)=\left\{ \begin{array}{cc} \rho_{0} & if\;a_{1}\in g_{1}C_{\left(\rho_{0},\eta_{0}\right)}\left(B_{t}\right)g_{1}^{-1}\\ \rho_{r} & if\;a_{1}\in g_{1}C_{\left(\rho_{i},\eta_{i}\right)}\left(B_{t}\right)g_{1}^{-1}↔g_{1}C_{\left(\rho_{i-1},\eta_{i-1}\right)}\left(B_{t}\right)g_{1}^{-1} \end{array}\right.$$

and

$$\nu_{B_{t}}\left(a_{1}\right)=\left\{ \begin{array}{cc} \eta_{0} & if\;a_{1}\in g_{1}C_{\left(\rho_{0},\eta_{0}\right)}\left(B_{t}\right)g_{1}^{-1}\\ \eta_{r} & if\;a_{1}\in g_{1}C_{\left(\rho_{i},\eta_{i}\right)}\left(B_{t}\right)g_{1}^{-1}↔g_{1}C_{\left(\rho_{i-1},\eta_{i-1}\right)}\left(B_{t}\right)g_{1}^{-1} \end{array}\right.,i=1,2,\ldots ,r\text{.}$$

Then $B_{t}\left(a_{1}\right)=A_{t}^{g_{1}}\left(a_{1}\right),\forall a_{1}\in G$ because i=1,2,…,r, we have

$$B_{t}\left(a_{1}\right)=\left(\rho_{i},\eta_{i}\right)$$

$$\Leftrightarrow a_{1}\in g_{1}C_{\left(\rho_{i},\eta_{i}\right)}\left(B_{t}\right)g_{1}^{-1}↔g_{1}C_{\left(\rho_{i-1},\eta_{i-1}\right)}\left(B_{t}\right)g_{1}^{-1}$$

$$\Leftrightarrow g_{1}^{-1}a_{1}g_{1}\in C_{\left(\rho_{i},\eta_{i}\right)}\left(B_{t}\right)↔C_{\left(\rho_{i-1},\eta_{i-1}\right)}\left(B_{t}\right)$$

$$\Leftrightarrow A_{t}\left(g_{1}^{-1}a_{1}g_{1}\right)=\left(\rho_{i},\eta_{i}\right)$$

$$\Leftrightarrow A_{t}^{g_{1}}\left(a_{1}\right)=\left(\rho_{i},\eta_{i}\right)$$

and

$$B_{t}\left(a_{1}\right)=\left(\rho_{0},\eta_{0}\right)$$

$$\Leftrightarrow a_{1}\in g_{1}C_{\left(\rho_{0},\eta_{0}\right)}\left(B_{t}\right)g_{1}^{-1}$$

$$\Leftrightarrow g_{1}^{-1}a_{1}g_{1}\in C_{\left(\rho_{0},\eta_{0}\right)}\left(B_{t}\right)$$

$$\Leftrightarrow A_{t}\left(g_{1}^{-1}a_{1}g_{1}\right)=\left(\rho_{0},\eta_{0}\right)$$

$$\Leftrightarrow A_{t}^{g_{1}}\left(a_{1}\right)=\left(\rho_{0},\eta_{0}\right)$$

Next, we define a map $I_{g_{1}}:G\to G$ by $I_{g_{1}}\left(a_{1}\right)=g_{1}^{-1}a_{1}g_{1},\forall a_{1}\in G\text{.}$ Then $I_{g_{1}}\in AutG\text{.}$ Since $A_{t}$ is a t-IFSG of G, it follows that $A_{t}^{g_{1}}=A_{t}\circ I_{g_{1}}=I_{g_{1}}^{-1}\left(A_{t}\right)$ is also a t-IFSG of G, as the inverse image of a t-IFSG by a group homomorphism is a t-IFSG. Hence $B_{t}=A_{t}^{g_{1}}$ is also a t-IFSG of G. Moreover, it is clear that $\Lambda \left(A_{t}^{g_{1}}\right)=\Lambda \left(A_{t}\right)$ and $C_{\left(\rho_{i},\eta_{i}\right)}\left(A_{t}^{g_{1}}\right)=g_{1}C_{\left(\rho_{i},\eta_{i}\right)}\left(A_{t}\right)g_{1}^{-1}$.

Thus, the chain of level subgroups of $A_{t}^{g_{1}}$ and hence of $B_{t}$ is given by

$$g_{1}C_{\left(\rho_{0},\eta_{0}\right)}\left(A_{t}\right)g_{1}^{-1}\subseteq g_{1}C_{\left(\rho_{1},\eta_{1}\right)}\left(A_{t}\right)g_{1}^{-1}\subseteq ...\subseteq g_{1}C_{\left(\rho_{r},\eta_{r}\right)}\left(A_{t}\right)g_{1}^{-1}=G$$

Example 7 Consider the 0.70−IFSG $A_{0.70}$ of $D_{4}$ as follows:

$$\mu_{A_{0.70}}\left(z_{1}\right)=\left\{ \begin{array}{cc} \begin{array}{c} 0.70\\ 0.65\\ 0.45 \end{array} & \begin{array}{c} if\;z_{1}=1\\ if\;z_{1}\in \left\{rs\right\}\\ if\;z_{1}\in \left\{s,r,r^{2},r^{3},r^{2}s,r^{3}s\right\} \end{array} \end{array}\right.$$

and

$$\nu_{A_{0.70}}\left(z_{1}\right)=\left\{ \begin{array}{cc} \begin{array}{c} 0.30\\ 0.35\\ 0.45 \end{array} & \begin{array}{c} if\;z_{1}=1\\ if\;z_{1}\in \left\{rs\right\}\\ if\;z_{1}\in \left\{s,r,r^{2},r^{3},r^{2}s,r^{3}s\right\} \end{array}. \end{array}\right.$$

In view of definition (3), we have

$$C_{\left(0.70,0.30\right)}\left(A_{0.70}\right)=\left\{1\right\}$$

$C_{\left(0.65,0.35\right)}\left(A_{0.70}\right)=\left\{1,rs\right\}$ and

$$C_{\left(0.45,0.45\right)}\left(A_{0.70}\right)=D_{4}\text{.}$$

Consider the 0.7−IFSG $B_{0.7}$ of $D_{4}$ as follows:

$$\mu_{B_{0.70}}\left(z_{1}\right)=\left\{ \begin{array}{cc} \begin{array}{c} 0.70\\ 0.65\\ 0.45 \end{array} & \begin{array}{c} if\;z_{1}=1\\ if\;z_{1}\in \left\{r^{3}s\right\}\\ if\;z_{1}\in \left\{s,r,r^{2},r^{3},rs,r^{2}s\right\} \end{array} \end{array}\right.$$

and

$$\nu_{B_{0.70}}\left(z_{1}\right)=\left\{ \begin{array}{cc} \begin{array}{c} 0.30\\ 0.35\\ 0.45 \end{array} & \begin{array}{c} if\;z_{1}=1\\ if\;z_{1}\in \left\{r^{3}s\right\}\\ if\;z_{1}\in \left\{s,r,r^{2},r^{3},rs,r^{2}s\right\} \end{array}. \end{array}\right.$$

In view of definition (3), we have

$$C_{\left(0.70,0.30\right)}\left(A_{0.70}\right)=\left\{1\right\}\text{,}$$

$$C_{\left(0.65,0.35\right)}\left(A_{0.70}\right)=\left\{1,rs\right\}$$

and

$$C_{\left(0.45,0.45\right)}\left(A_{0.70}\right)=D_{4}\text{.}$$

Similarly, $C_{\left(0.70,0.30\right)}\left(B_{0.70}\right)=\left\{1\right\}$, $C_{\left(0.65,0.35\right)}\left(B_{0.70}\right)=\left\{1,r^{3}s\right\}$ and

$$C_{\left(0.45,0.45\right)}\left(B_{0.70}\right)=D_{4}\text{.}$$

The chain of level subgroups of 0.70−IFSG $A_{0.70}$ and $B_{0.70}$ of $D_{4}$ is given as:

$$C_{\left(0.70,0.30\right)}\left(A_{0.70}\right)\subseteq C_{\left(0.65,0.35\right)}\left(A_{0.70}\right)\subseteq C_{\left(0.45,0.45\right)}\left(A_{0.70}\right)$$

and

$$C_{\left(0.70,0.30\right)}\left(B_{0.70}\right)\subseteq C_{\left(0.65,0.35\right)}\left(B_{0.70}\right)\subseteq C_{\left(0.45,0.45\right)}\left(B_{0.70}\right)$$

In view of Theorem (24), we have

$$aC_{\left(0.70,0.30\right)}\left(B_{0.7}\right)a^{-1}\subseteq aC_{\left(0.65,0.35\right)}\left(B_{0.7}\right)a^{-1}\subseteq aC_{\left(0.45,0.45\right)}\left(B_{0.7}\right)a^{-1}\text{.}$$

Thus $B_{0.7}=A_{0.7}^{a}$.

Theorem 25 Let $A_{t}$ be a t-IFSG $B_{t}$ of a finite group G then the number of distinct t-IFCSG of $A_{t}$ is equal to the index of its normalizer in G.

**Proof.** Let $A_{t}$ be a t-IFSG of a finite group G. Consider the decomposition of G as a union of left cosets of $N\left(A_{t}\right)$:

(11) $$G=a_{1}N\left(A_{t}\right)\cup a_{2}N\left(A_{t}\right)\cup ...\cup a_{k}N\left(A_{t}\right)$$

where k is the number of distinct left cosets, that is, the index of $N\left(A_{t}\right)$ in G.

Let $a\in N\left(A_{t}\right)$ and choose i such that 1≤i≤k. Then for any $a_{i}\in G$.

$$\mu_{A_{t}}^{a_{i}a}\left(b_{1}\right)=\mu_{A_{t}}\left({\left(a_{i}a\right)}^{-1}b_{1}\left(a_{i}a\right)\right)$$

$$=\mu_{A_{t}}\left(a^{-1}\left(a_{i}^{-1}b_{1}a_{i}\right)a\right)$$

$$=\mu_{A_{t}}^{a}\left(a_{i}^{-1}b_{1}a_{i}\right)$$

$$=\mu_{A_{t}}\left(a_{i}^{-1}b_{1}a_{i}\right)$$

$$=\mu_{A_{t}}^{a_{i}}\left(b_{1}\right)$$

This means that $\mu_{A_{t}}^{a_{i}a}=\mu_{A_{t}}^{a_{i}}\forall a\in N\left(A_{t}\right),1\leq i\leq k$.

Similarly, $\nu_{A_{t}}^{a_{i}a}=\nu_{A_{t}}^{a_{i}}\forall a\in N\left(A_{t}\right),1\leq i\leq k$.

So, any two elements of G which lies in the same cosets $a_{i}N\left(A_{t}\right)$ give rise to the same $t-IFCSG\;A_{t}^{a_{i}}$ of $A_{t}\text{.}$ Now we show that two distinct t-IFCSG of $A_{t}\text{.}$ Suppose that

(12) $$A_{t}^{a_{i}}=A_{t}^{a_{j}}$$

where i≠j and 1≤i,j≤k. Now relation (12) implies that

$$A_{t}^{a_{i}}\left(b_{1}\right)=A_{t}^{a_{j}}\left(b_{1}\right)\forall b_{1}\in G$$

This implies that $A_{t}\left(a_{i}^{-1}b_{1}a_{i}\right)=A_{t}\left(a_{j}^{-1}b_{1}a_{j}\right),\forall b_{1}\in G$.

Substituting $b_{1}=a_{j}c_{1}a_{j}^{-1}$ in the above relation, we have

$$A_{t}\left(a_{i}^{-1}a_{j}c_{1}a_{j}^{-1}a_{i}\right)=A_{t}\left(c_{1}\right),\forall c_{1}\in G$$

This further implies that $A_{t}\left(\left(a_{i}^{-1}a_{j}\right)c_{1}\left(\;a_{j}^{-1}a_{i}\right)\right)=A_{t}\left(c_{1}\right),\forall c_{1}\in G$.

This further implies that $A_{t}^{a_{j}^{-1}a_{i}}\left(c_{1}\right)=A_{t}\left(c_{1}\right),\forall c_{1}\in G\text{.}$ This means that $a_{j}^{-1}a_{i}\in N\left(A_{t}\right)$.

Consequently, $a_{i}N\left(A_{t}\right)=a_{j}N\left(A_{t}\right)\text{.}$ However, if i≠j this is not possible as (11) represents a partition of G into pairwise disjoint cosets. Hence the number of distinct conjugates of $A_{t}$ is equal to the index of $N\left(A_{t}\right)$ in G.

Definition 20 Let $A_{t}$ be a t-IFSG of a group G and $a_{1}\in G\text{.}$ Then $a_{1}^{-1}\;C_{\left(\rho ,\eta \right)}\left(A_{t}\right)\;a_{1}$ forms a t-IFCSG G. This t-IFSG is called t-IFCSG to $A_{t}\text{.}$ The set

$$Cl\left(A_{t}\right)=\left\{a_{1}^{-1}\;C_{\left(\rho ,\eta \right)}\left(A_{t}\right)\;a_{1}:a_{1}\in G\right\}$$

is called the class of t-IFCSG to $A_{t}$.

Example 8 Consider the t-IFSG of the dihedral group $D_{4}$ for the value t=0.70 as follows:

$$\mu_{A_{0.70}}\left(z_{1}\right)=\left\{ \begin{array}{cc} \begin{array}{c} 0.70\\ 0.50\\ 0.40 \end{array} & \begin{array}{c} if\;z_{1}=1\\ if\;z_{1}=rs\\ if\;z_{1}\in \left\{r,r^{2},r^{3},s,r^{2}s,r^{3}s\right\} \end{array} \end{array}\right.$$

and

$$\nu_{A_{0.70}}\left(z_{1}\right)=\left\{ \begin{array}{cc} \begin{array}{c} 0.30\\ 0.40\\ 0.50 \end{array} & \begin{array}{c} if\;z_{1}=1\\ if\;z_{1}=rs\\ if\;z_{1}\in \left\{r,r^{2},r^{3},s,r^{2}s,r^{3}s\right\} \end{array}. \end{array}\right.$$

In the light of definition (2), we acquire

$$C_{\left(0.50,0.40\right)}\left(A_{0.70}\right)=\left\{1,rs\right\}$$

The required class of 0.70−IFCSG to $A_{0.70}$ is obtained as

$$Cl\left(A_{0.70}\right)=\left\{H_{1},H_{2},H_{3},H_{4}\right\}\text{,}$$

where $H_{1}=\left\{1,rs\right\},H_{2}=\left\{1,s\right\},H_{3}=\left\{1,r^{2}s\right\}$ and $H_{4}=\left\{1,r^{3}s\right\}$.

Corollary 7 Let $a_{1}^{-1}\;C_{\left(\rho ,\eta \right)}\left(A_{t}\right)\;a_{1}$ be t-IFCSG to $A_{t}$ then $O\left[Cl\left(A_{t}\right)\right]=\frac{O\left(G\right)}{O\left[N\left(A_{t}\right)\right]}$**.**

The following illustration establishes the algebraic facts discussed in the above result.

Example 9 The application of the definition (13) and corollary (7) gives that: $O\left[Cl\left(A_{0.70}\right)\right]=\frac{8}{2}=4\text{.}$ (See example 8).

## 6 t-Intuitionistic fuzzification of Sylow's theorems

In this section, we define the notion of t-intuitionistic fuzzy Sylow p− subgroup of finite group G. Moreover, we prove the t-intuitionistic fuzzy version of Sylow's Theorems.

Definition 21 A t-IFSG $A_{t}$ of a group, G is called a t-intuitionistic fuzzy Sylow's p− subgroup $\left(t-lF{Syl}_{p}\right)$ if the support set $A_{t}^{*}$ is a Sylow's p− subgroup of G.

Definition 22 Let $A_{t}$ be a t-IFSG of a finite group G. A t-IFSG $A_{t}$ is called t-intuitionistic fuzzy Sylow's p− subgroup$\left(t-lF{Syl}_{p}\right)$ of group G, if one of the level subgroups of $A_{t}$ is Sylow's p− subgroup of G.

There may be two or more level subgroups $A_{t}$ which are Sylow p− subgroup, but the support of $A_{t}$ may not be Sylow p− subgroup of G. However, definition (20) implies definition (21).

The following example illustrates the existence of the Sylow p− subgroup of a $t-lF{Syl}_{p}\left(G\right)$.

Example 10 Consider the direct product of the groups: $G=<r:r^{5}=1>$ and $H=<s:s^{2}=1>$ as G×H={(r,s):r∈G,s∈H}.

Consider the 0.70−IFSG of G×H is given by:

$$\mu_{A_{0.70}}\left(z_{1}\right)=\left\{ \begin{array}{cc} \begin{array}{c} 0.70\\ 0.50\\ 0.40 \end{array} & \begin{array}{c} if\;z_{1}=\left(1,1\right)\\ if\;z_{1}\in <\left(r,1\right)>-\left\{\left(1,1\right)\right\}\\ if\;z_{1}\in G\times H-<\left(r,1\right)> \end{array} \end{array}\right.$$

and

$$\nu_{A_{0.70}}\left(z_{1}\right)=\left\{ \begin{array}{cc} \begin{array}{c} 0.30\\ 0.40\\ 0.60 \end{array} & \begin{array}{c} if\;z_{1}=\left(1,1\right)\\ if\;z_{1}\in <\left(r,1\right)>-\left\{\left(1,1\right)\right\}\\ if\;z_{1}\in G\times H-<\left(r,1\right)> \end{array} \end{array}\right.$$

In view of definition (3), we have

$${Ĉ}_{\left(0.5,0.4\right)}\left(A_{0.70}\right)=\left\langle \left(r,1\right)\right\rangle \text{,}$$

which is a Sylow 5-subgroup of G×H.

Thus, $A_{0.70}$ is a $t-lF{Syl}_{5}$ of G×H.

The following result describes the t-intuitionistic fuzzy version of Sylow's First Theorem.

Theorem 26 **(t-Intuitionistic Fuzzification of Sylow's First Theorem)**. Let $A_{t}$ be a t-IFSG of a group G such that $O\left(G\right)=p^{k}m$ where p is a prime and k,m are positive integers with (p,m)=1. Assume that p divides $A_{t}^{*}=H\text{.}$ Then there exists a $t-IF{Syl}_{p}\;B_{t}$ of G such that $B_{t}\subseteq A_{t}$.

**Proof.** If H is a Sylow p-subgroup, then there is nothing to prove. Assume that H is not a Sylow p-subgroup of G. Let $\rho =\mathrm{Min}\left\{\mu_{A_{t}}\left(a_{1}\right):\mu_{A_{t}}\left(a_{1}\right)>0,a_{1}\in G\right\}$ and $\eta =\mathrm{Max}\left\{\nu_{A_{t}}\left(a_{1}\right):\nu_{A_{t}}\left(a_{1}\right)>0,a_{1}\in G\right\}\text{.}$ Clearly ρ≠0 and η≠1. Since G is finite, therefore, $C_{\left(\rho ,\eta \right)}\left(A_{t}\right)=A_{t}^{*}=H\text{.}$ Since p divides the order of H so by Sylow's first theorem, there exists a Sylow p− subgroup L of H. By our assumption, $O\left(L\right)=p^{l}$ where 1≤l≤k. Further, L will be contained in a subgroup $L^{\prime }$ of G. Consider a $t-IFSB_{t}$ of G as follows:

$\mu_{B_{t}}\left(z_{1}\right)=\left\{ \begin{array}{cc} \begin{array}{c} 1\\ \rho \end{array} & \begin{array}{c} if\;z_{1}=e\\ if\;z_{1}\in L-\left\{e\right\} \end{array}\\ \begin{array}{c} \rho^{\prime }\\ 0 \end{array} & \begin{array}{c} if\;z_{1}\in L^{\prime }-L\\ if\;z_{1}\in G-L^{\prime } \end{array} \end{array}\right.$ and $\nu_{B_{t}}\left(z_{1}\right)=\left\{ \begin{array}{cc} \begin{array}{c} 0\\ \eta \end{array} & \begin{array}{c} if\;z_{1}=e\\ if\;z_{1}\in L-\left\{e\right\} \end{array}\\ \begin{array}{c} \eta^{\prime }\\ 1 \end{array} & \begin{array}{c} if\;z_{1}\in L^{\prime }-L\\ if\;z_{1}\in G-L^{\prime } \end{array} \end{array}\right.$.where $0<\rho^{\prime },\eta^{\prime }<1\text{.}$ Note that $B_{t}$ is t-IFSG of G such that $B_{t}\subseteq A_{t}$. Moreover, in view of definition (21), we have $B_{t}$ is a $t-IF{Syl}_{p}$ of G as ${Ĉ}_{\left(\rho ,\eta \right)}\left(B_{t}\right)=L$.

Theorem 27 A t-IFCSG of $t-IF{Syl}_{p}$ of a group G is a $t-IF{Syl}_{p}$ of G.

**Proof.** Let $A_{t}$ be a $t-IF{Syl}_{p}$ of a group G and $B_{t}$ is t-IFC to $A_{t}$ of G. In view of definition (19), we have ${Ć}_{\left(\rho ,\eta \right)}\left(B_{t}\right)=a_{1}^{-1}\;{Ć}_{\left(\rho ,\eta \right)}\left(A_{t}\right)a_{1},a_{1}\in G\forall \rho ,\eta$. This implies that $B_{t}^{*}=a_{1}^{-1}A_{t}^{*}a_{1}\text{.}$ Application of the Theorem (26) and using the fact that $A_{t}$ is $t-IF{Syl}_{p}$ of G, we have a Sylow p-subgroup H of G contained in $A_{t}^{*}\text{.}$ Moreover, in view of Sylow's Second Theorem, $a_{1}^{-1}Ha_{1}$ being a conjugate of H is itself a Sylow p-subgroup of G. Further, $a_{1}^{-1}Ha_{1}$ is contained in $a_{1}^{-1}A_{t}^{*}a_{1}$ and so $B_{t}$ is a $t-IF{Syl}_{p}$ of G.

Remark 4 Two distinct $t-IF{Syl}_{p}$ need not be t-IFC to each other.

We describe the above algebraic aspect in the subsequent example.

Example 11 Consider the Sylow 2− subgroup $H_{1}$ and $H_{2}$ of $S_{5}$ as follows: $H_{1}=<\left(12\right)\left(35\right),\left(1325\right)>$ and $H_{2}=<\left(24\right)\left(35\right),\left(2345\right)>$.

The $0.70-IFSG\;A_{0.70}$ and $B_{0.7}$ of $S_{5}$ as follows:

$$\mu_{A_{0.70}}\left(z_{1}\right)=\left\{ \begin{array}{cc} \begin{array}{c} 0.70\\ 0.65\\ 0.60 \end{array} & \begin{array}{c} if\;z_{1}=1\\ if\;z_{1}\in \left\langle \left(24\right)\left(35\right)\right\rangle -\left\{1\right\}\\ if\;z_{1}\in \left\langle \left(24\right),\left(35\right)\right\rangle -\left\langle \left(24\right)\left(35\right)\right\rangle \end{array}\\ \begin{array}{c} 0.50\\ 0.30 \end{array} & \begin{array}{c} if\;z_{1}\in \left\langle \left(25\right)\left(34\right),\left(2345\right)\right\rangle -\left\langle \left(24\right),\left(35\right)\right\rangle \\ otherwise \end{array} \end{array}\right.$$

and

$$\nu_{A_{0.70}}\left(z_{1}\right)=\left\{ \begin{array}{cc} \begin{array}{c} 0.30\\ 0.35\\ 0.40 \end{array} & \begin{array}{c} if\;z_{1}=1\\ if\;z_{1}\in \left\langle \left(24\right)\left(35\right)\right\rangle -\left\{1\right\}\\ if\;z_{1}\in \left\langle \left(24\right),\left(35\right)\right\rangle -\left\langle \left(24\right)\left(35\right)\right\rangle \end{array}\\ \begin{array}{c} 0.50\\ 0.60 \end{array} & \begin{array}{c} iif\;z_{1}\in \left\langle \left(25\right)\left(34\right),\left(2345\right)\right\rangle -\left\langle \left(24\right),\left(35\right)\right\rangle \\ otherwise \end{array} \end{array}\right.$$

and

$$\mu_{B_{0.70}}\left(z_{1}\right)=\left\{ \begin{array}{cc} \begin{array}{c} 0.70\\ 0.65\\ 0.60 \end{array} & \begin{array}{c} if\;z_{1}=1\\ if\;z_{1}\in \left\langle \left(12\right)\left(35\right)\right\rangle -\left\{1\right\}\\ if\;z_{1}\in \left\langle \left(1325\right)\right\rangle -\left\langle \left(12\right)\left(35\right)\right\rangle \end{array}\\ \begin{array}{c} 0.50\\ 0.30 \end{array} & \begin{array}{c} if\;z_{1}\in \left\langle \left(12\right)\left(35\right),\left(1325\right)\right\rangle -\left\langle \left(1325\right)\right\rangle \\ otherwise \end{array} \end{array}\right.$$

and

$$\nu_{B_{0.70}}\left(z_{1}\right)=\left\{ \begin{array}{cc} \begin{array}{c} 0.30\\ 0.35\\ 0.40 \end{array} & \begin{array}{c} if\;z_{1}=1\\ if\;z_{1}\in \left\langle \left(12\right)\left(35\right)\right\rangle -\left\{1\right\}\\ if\;z_{1}\in \left\langle \left(1325\right)\right\rangle -\left\langle \left(12\right)\left(35\right)\right\rangle \end{array}\\ \begin{array}{c} 0.50\\ 0.60 \end{array} & \begin{array}{c} if\;z_{1}\in \left\langle \left(12\right)\left(35\right),\left(1325\right)\right\rangle -\left\langle \left(1325\right)\right\rangle \\ otherwise \end{array} \end{array}\right.$$

Clearly $A_{0.70}$ and $B_{0.70}$ are $0.70-IF{Syl}_{2}$ of $S_{5}$.

Moreover, $A_{0.70}$ is not 0.7−IFC to $B_{0.70}$ because the level subgroups: ${Ć}_{\left(0.6,0.4\right)}\left(A_{0.70}\right)=<\left(24\right),\left(35\right)>$ is noncyclic and ${Ć}_{\left(0.6,0.4\right)}\left(B_{0.70}\right)=<\left(1325\right)>$ is cyclic.

The following Theorem describes a t-intuitionistic fuzzy version of Sylow’s Second Theorem.

Theorem 28 **(t-Intuitionistic Fuzzification of Sylow Second Theorem)**. For any two $t-lF{Syl}_{p}$$A_{t}$ and $B_{t}$ having the same level subgroups such that ${Ć}_{\left(\rho ,\eta \right)}\left(B_{t}\right)=a_{1}^{-1}\;{Ć}_{\left(\rho ,\eta \right)}\left(A_{t}\right)a_{1}\text{,}$ for some fixed $a_{1}\in G\text{,}$ then $A_{t}$ and $B_{t}$ are t−IFC to each other.

**Proof.** Let ${Ć}_{\left(\rho ,\eta \right)}\left(B_{t}\right)=a_{1}^{-1}\;{Ć}_{\left(\rho ,\eta \right)}\left(A_{t}\right)a_{1}\text{.}$ Assume that $A_{t}$ and $B_{t}$ are not t-IFC to each other it follows that $\mu_{A_{t}}\left(b_{1}\right)>\mu_{B_{t}}\left(a_{1}^{-1}b_{1}a_{1}\right)$ and $\nu_{A_{t}}\left(b_{1}\right)<\nu_{B_{t}}\left(a_{1}^{-1}b_{1}a_{1}\right)\text{,}$ for $a_{1},b_{1}\in G$ (13)

Then $b_{1}\in {Ć}_{\left(\rho ,\eta \right)}\left(B_{t}\right)$ and $a_{1}^{-1}b_{1}a_{1}\notin {Ć}_{\left(\rho ,\eta \right)}\left(A_{t}\right)$. This means that $b_{1}\in a_{1}^{-1}{Ć}_{\left(\rho ,\eta \right)}\left(A_{t}\right)a_{1}$ and $a_{1}^{-1}b_{1}a_{1}\notin {Ć}_{\left(\rho ,\eta \right)}\left(A_{t}\right)$.

This implies that $a_{1}b_{1}a_{1}^{-1}\in {Ć}_{\left(\rho ,\eta \right)}\left(A_{t}\right)$ and $a_{1}^{-1}b_{1}a_{1}\notin {Ć}_{\left(\rho ,\eta \right)}\left(A_{t}\right)\text{.}$ This further implies that ${\left(a_{1}^{-1}b_{1}a_{1}\right)}^{-1}\in {Ć}_{\left(\rho ,\eta \right)}\left(A_{t}\right)$ and $a_{1}^{-1}b_{1}a_{1}\notin {Ć}_{\left(\rho ,\eta \right)}\left(A_{t}\right)$.

This means that $c^{-1}\in {Ć}_{\left(\rho ,\eta \right)}\left(A_{t}\right)$ and $c\notin {Ć}_{\left(\rho ,\eta \right)}\left(A_{t}\right)$.

This shows that ${Ć}_{\left(\rho ,\eta \right)}\left(A_{t}\right)$ is not a subgroup of G.

Which is a contradiction against relation (13).

Hence, $\mu_{A_{t}}\left(b_{1}\right)=\mu_{B_{t}}\left(a_{1}^{-1}b_{1}a_{1}\right)$ and $\nu_{A_{t}}\left(b_{1}\right)=\nu_{B_{t}}\left(a_{1}^{-1}b_{1}a_{1}\right)\text{,}$ $\forall a_{1},b_{1}\in G$.

We conclude that $A_{t}$ and $B_{t}$ are t-IFC to each other.

Remark 5 Let $A_{t}$ and $B_{t}$ be any two $t-lF{Syl}_{p}$ of a group G and $\Lambda \left(A_{t}\right)=\Lambda \left(B_{t}\right)$ then $A_{t}$ and $B_{t}$ are t-IFC to each other.

The following Theorem describes the t-intuitionistic fuzzy version of Sylow's third Theorem.

Theorem 29 **(t-Intuitionistic Fuzzification of Sylow Third Theorem)**. The number of distinct $t-lF{Syl}_{p}$ of a finite group G is equal to the index of the normalizer of $t-IF{Syl}_{p}$ $A_{t}$ in G.

**Proof.** In view of corollary (7) and using the fact that $A_{t}$ is a $t-IF{Syl}_{p}$, we have

$$O\left[Cl\left(A_{t}\right))\right]=\frac{O\left(G\right)}{O\left[N\left(A_{t}\right)\right]}$$

Consider the collection of all $t-lF{Syl}_{p}$ conjugate to $A_{t}$ has

$$Cl({Ć}_{\left(\rho ,\eta \right)}\left(A_{t}\right)=\left\{{Ć}_{\left(\rho ,\eta \right)}\left(B_{t}\right):a_{1}^{-1}\;{Ć}_{\left(\rho ,\eta \right)}\left(A_{t}\right)a_{1}={Ć}_{\left(\rho ,\eta \right)}\left(B_{t}\right),a_{1}\in G\right\}$$

Consequently, the number of $t-lF{Syl}_{p}$ of G is $\frac{O\left(G\right)}{O\left[N\left(A_{t}\right)\right]}$.

We illustrate the above algebraic fact in the following example.

Example 12 Consider the finite presentation of the dihedral group $D_{5}$ of order 10 as follows:

$$D_{5}=<r,s:r^{5}=s^{2}=1,sr=r^{4}s>$$

The IFSG of $D_{5}$ is defined as follows: $\mu_{A}\left(z_{1}\right)=\left\{ \begin{array}{cc} \begin{array}{c} 1\\ 0.60\\ 0.40 \end{array} & \begin{array}{c} if\;z_{1}=1\\ if\;z_{1}\in <r>-\left\{1\right\}\\ if\;z_{1}\in D_{5}-<r> \end{array} \end{array}\right.$ and $\nu_{A}\left(z_{1}\right)=\left\{ \begin{array}{cc} \begin{array}{c} 0\\ 0.40\\ 0.50 \end{array} & \begin{array}{c} if\;z_{1}=1\\ if\;z_{1}\in <r>-\left\{1\right\}\\ if\;z_{1}\in D_{5}-<r> \end{array} \end{array}\right.$.

The t−IFSG $A_{t}$ of $D_{5}$ corresponding to the value t=0.70 is given by: $\mu_{A_{0.70}}\left(z_{1}\right)=\left\{ \begin{array}{cc} \begin{array}{c} 0.70\\ 0.60\\ 0.40 \end{array} & \begin{array}{c} if\;z_{1}=1\\ if\;z_{1}\in <r>-\left\{1\right\}\\ if\;z_{1}\in D_{5}-<r> \end{array} \end{array}\right.$ and $\nu_{A_{0.70}}\left(z_{1}\right)=\left\{ \begin{array}{cc} \begin{array}{c} 0.30\\ 0.40\\ 0.50 \end{array} & \begin{array}{c} if\;z_{1}=1\\ if\;z_{1}\in <r>-\left\{1\right\}\\ if\;z_{1}\in D_{5}-<r> \end{array} \end{array}\right.$.

By using definition (3), we have

$$C_{\left(0.6,0.4\right)}\left(A_{0.70}\right)=<r>,$$

which is a $0.70-lF{Syl}_{5}$ of $D_{5}$.

The class of $0.70-lF{Syl}_{5}$ of $D_{5}$ is

$$\mathrm{Cl}\left(<r>\right)=<r>.$$

The 0.70− intuitionistic fuzzy normalizer of $0.70-IF{Syl}_{5}$ of $D_{5}$ is

$$N\left(A_{0.70}\right)=D_{5}.$$

In view of Theorem (29), we have only one $0.70-lF{Syl}_{5}$ of $A_{0.70}$ in $D_{5}$.

The subsequent results show that a t-intuitionistic fuzzy normal Sylow p− subgroup of a G is unique.

Theorem 30 Let $A_{t}$ and $B_{t}$ be any two $t-lF{Syl}_{p}$ of a group G. If $A_{t}$ is t-IFSG of a group G, then $A_{t}↔B_{t}$. Moreover, if ${\Lambda (A}_{t})=\Lambda \left(B_{t}\right)$ then $A_{t}=B_{t}\text{.}$

**Proof.** If O(G) is a power of p, then the case is obvious. Assume that O(G) is not a power of p.

Consider $\mu_{A_{t}}\left(e\right)=\rho ,\nu_{A_{t}}\left(e\right)=\eta$ and $\mu_{B_{t}}\left(e\right)=\rho^{\prime },\nu_{B_{t}}\left(e\right)=\eta^{\prime }$. Then ${Ć}_{\left(\rho ,\eta \right)}\left(A_{t}\right)$ and ${Ć}_{\left(\rho ,\eta \right)}\left(B_{t}\right)$ are $t-IF{Syl}_{p}$ of G. Since $A_{t}$ is a t−IFNSG. By using Theorem (2), we have ${Ć}_{\left(\rho ,\eta \right)}\left(A_{t}\right)$ is a normal subgroup of G. Consequently, ${Ć}_{\left(\rho ,\eta \right)}\left(A_{t}\right)$ =${Ć}_{\left(\rho ,\eta \right)}\left(B_{t}\right)$. This shows that $\Lambda \left(A_{t}\right)=\Lambda (B_{t})$). We obtain that $A_{t}↔B_{t}$. From Theorem (19), we get $A_{t}=B_{t}$.

In the following Theorem, we establish a unique $t-IF{Syl}_{p}$ of G is a t-IFNSG of group G.

Theorem 31 For any $t-IF{Syl}_{p}$ $A_{t}$ of a group G. If $A_{t}∼B_{t}$ $\forall B_{t}\in \Omega$ where Ω is the collection of all $t-IF{Syl}_{p}$ of group G then $A_{t}$ is a t−IFNSG of G.

**Proof.** According to the Theorem (2), we obtain that ${Ć}_{\left(\rho ,\eta \right)}\left(A_{t}\right)$ is a normal subgroup of G and the fact that $\Lambda \left(A_{t}\right)=\Lambda \left(B_{t}\right),\forall B_{t}\in \Omega$.

## 7 Conclusion and future scopes

The t-intuitionistic fuzzy form of Sylow's theorem may be applied in real-world situations with subgroup analysis uncertainty, ambiguity, and imprecision. This approach improves decision-making, data analysis, optimization, and social network analysis, allowing for a complete knowledge of real-life scenarios with unpredictable group structures. In this article, we have introduced the concept of t− IFC element. After, we initiated the t-intuitionistic fuzzy conjugacy classes of t-IFSG. Moreover, the fundamental algebraic attributes of these phenomena were derived. Additionally, the class equation for t-IFSG was explained, along with an example. This paper has established the theory to formulate the t-intuitionistic fuzzy version of Sylow's theorems of t-IFSG of a finite group.

The theory presented in this article can be generalized to high-level uncertain environments, specifically t-picture fuzzy environments, t-spherical fuzzy environments, t-linear Diophantine fuzzy environments, and many more. Furthermore, we will apply the concept of t− IFSG in the fields of cryptography and image processing in our future work.

## Author contribution statement

Laila Latif: Analyzed and interpreted the data; Contributed reagents, materials, analysis tools or data; wrote the paper.

Umer Shuaib: Conceived and designed the experiments; Performed the experiments; Analyzed and interpreted the data.

## Additional information

No additional information is available for this paper.

## Data availability statement

Data included in article/supplementary material/referenced in article.

## Declaration of competing interest

The authors declare that they have no known competing financial interests or personal relationships that could have appeared to influence the work reported in this paper.
